# Simulating within host human immunodeficiency virus 1 genome evolution in the persistent reservoir

**DOI:** 10.1093/ve/veaa089

**Published:** 2020-11-23

**Authors:** Bradley R Jones, Jeffrey B Joy

**Affiliations:** BC Centre for Excellence in HIV/AIDS, 608-1081 Burrard Street, Vancouver, BC V6Z 1Y6, Canada; Bioinformatics Program, University of British Columbia, 100-570 West 7th Avenue, Vancouver, BC V5T 4S6, Canada; BC Centre for Excellence in HIV/AIDS, 608-1081 Burrard Street, Vancouver, BC V6Z 1Y6, Canada; Bioinformatics Program, University of British Columbia, 100-570 West 7th Avenue, Vancouver, BC V5T 4S6, Canada; Department of Medicine, University of British Columbia, 2775 Laurel Street, 10th Floor, Vancouver, BC V5Z 1M9, Canada

**Keywords:** simulation, human immunodeficiency virus 1, viral latency, fitness

## Abstract

The complexities of viral evolution can be difficult to elucidate. Software simulating viral evolution provides powerful tools for exploring hypotheses of viral systems, especially in situations where thorough empirical data are difficult to obtain or parameters of interest are difficult to measure. Human immunodeficiency virus 1 (HIV-1) infection has no durable cure; this is primarily due to the virus’ ability to integrate into the genome of host cells, where it can remain in a transcriptionally latent state. An effective cure strategy must eliminate every copy of HIV-1 in this ‘persistent reservoir’ because proviruses can reactivate, even decades later, to resume an active infection. However, many features of the persistent reservoir remain unclear, including the temporal dynamics of HIV-1 integration frequency and the longevity of the resulting reservoir. Thus, sophisticated analyses are required to measure these features and determine their temporal dynamics. Here, we present software that is an extension of SANTA-SIM to include multiple compartments of viral populations. We used the resulting software to create a model of HIV-1 within host evolution that incorporates the persistent HIV-1 reservoir. This model is composed of two compartments, an active compartment and a latent compartment. With this model, we compared five different date estimation methods (Closest Sequence, Clade, Linear Regression, Least Squares, and Maximum Likelihood) to recover the integration dates of genomes in our model’s HIV-1 reservoir. We found that the Least Squares method performed the best with the highest concordance (0.80) between real and estimated dates and the lowest absolute error (all pairwise *t* tests: *P* < 0.01). Our software is a useful tool for validating bioinformatics software and understanding the dynamics of the persistent HIV-1 reservoir.

## 1. Introduction

Virus evolution is complex, from the population dynamics of epidemic spread to the complexities of the spread of a virus throughout the body of an individual host. Phylodynamic methods are essential for understanding these viral systems at all levels of epidemic hierarchies (from population to individual infection). However, such tools must be robustly tested before we have confidence in their efficacy to reconstruct viral evolution. A reliable way to test phylodynamic methods is to apply the tools to data sets where the results are confidently known. Since it is typically difficult to be certain of results derived from empirical data, it is imperative to validate phylodynamic methods on simulated data where inferences of parameter estimates can be compared with the truth.

Human immunodeficiency virus 1 (HIV-1) infection can currently be managed via combination antiretroviral therapy (cART) by halting viral replication thereby lowering plasma viral load, prolonging progression to acquired immune deficiency syndrome (AIDS) and minimizing transmission risk (Hogg 1998; Pallela et al. 1998). However, a durable HIV-1 cure cannot be achieved through cART alone since cART cannot (currently) target proviruses integrated into the host’s genome in a transcriptionally latent state (Chun et al. 1997; Finzi 1997; Finzi et al. 1999). HIV-1 proviruses from this HIV-1 persistent reservoir can reactivate years or decades after integration to produce replication-competent virions, meaning that cessation from cART can result in viral rebound and continuation of active infection after a few weeks (Davey et al. 1999). For this reason, a durable HIV-1 cure must eliminate or permanently suppress every copy of HIV-1 integrated in the host’s cells.

Crucial gaps in our knowledge of the HIV-1 reservoir persist, including the rate of introduction, genetic persistence and the specific timing of integration of the HIV-1 reservoir. For example, it is currently debated whether the reservoir is contributed to and maintained throughout the course of infection (Jones et al. 2018, 2020; Brooks et al. 2020) or if a high turnover during active infection results in a reservoir containing younger viruses (Brodin et al. 2016; Abrahams et al. 2019). In order to address these knowledge gaps, we must develop and employ sophisticated phylodynamic tools, and these tools need to be assessed and validated to ensure their accuracy and efficacy. One means of validation is *in silico* simulation, but there are, to our knowledge, no tools currently available that are specifically designed to simulate HIV-1 genomes within host accommodating the HIV-1 reservoir. Current genome simulation software (Laval and Excoffier 2004; Mailund et al. 2005; Rodriguez-Carvajal 2008; Fletcher and Yang 2009; Petitjean and Vanet 2014; Haller and Messer 2017; Jariani et al. 2019) do not incorporate fitness, can only simulate one compartment/deme, or do not simulate viral replication, but instead assume bisexual reproduction with diploid genomes. The ability to simulate multiple compartments is necessary for HIV-1 within host simulation because of the presence the HIV-1 reservoir which acts as a separate compartment with different evolutionary characteristics than virus undergoing active replication.

We present software able to simulate genome evolution with multiple compartments. Within this software, we created a simulation model specifically designed to simulate HIV-1 genome evolution within host that incorporates the dynamics of the HIV-1 reservoir. As a case study, we applied five different date estimation methods to data derived from our model to compare and evaluate the accuracy of these methods in recovering integration dates of proviral genomes.

## 2. Extending SANTA-SIM

The Java software, SANTA-SIM (Jariani et al. 2019), is a forward-time evolution simulator, which simulates viral genomes in a population. Generations occur in a stepwise fashion where the genomes mutate based on a substitution matrix and replicate based on population growth models (such as fixed-size population, exponential/logistic growth, and dynamic population growth) and selection (such as purifying selection, population size-dependent fitness, and other allele-based selection). Sampling of the population can be done periodically or at specified generations to retrieve genomes or phylogenies. SANTA-SIM also includes genome insertions, deletions, and recombination, in addition to gene mapping. The simulation runs over one or more epochs where the model parameters: mutation, population growth, fitness, sampling, etc., can change. Model parameters are specified in an XML file, which allows the modification of any of the parameters mentioned above in addition to a starting genome. This is akin to other bioinformatic software such as BEAST (Suchard et al. 2018; Bouckaert et al. 2019).

However, SANTA-SIM is restricted to working within a single population/compartment with a singular mutation rate, population dynamics, and fitness landscape per epoch. This is not adequate for modelling within host HIV-1 evolution where there is one population of viruses, which is constantly replicating, mutating, and evolving and another population, the HIV-1 persistent reservoir, which is in a latent state. To overcome this limitation, we modified SANTA-SIM to facilitate multiple compartments of viruses and genome transfer between compartments. We chose to modify SANTA-SIM over other software or creating new software due to its ease of extensibility, breadth of currently available model features, and mutability via the XML specification file. We added Java classes to represent compartments of viruses and epochs for compartments, and we added a Java interface for genome transfer with inheriting classes to model gene flow between compartments by a rate probability matrix, fitness or a timed event. Reduced class diagrams of SANTA-SIM and our modified SANTA-SIM are shown in Supplementary Figure S1 highlighting our changes. Users can specify compartments for their simulation model by adding a Compartment element to their XML file for each compartment they wish to include. Our modifications also maintain backwards compatibility with the original SANTA-SIM in that XML files without a Compartment element can be read and behave as if they had one compartment.

## 3. Simulation of the HIV-1 persistent reservoir

**Figure 1.**
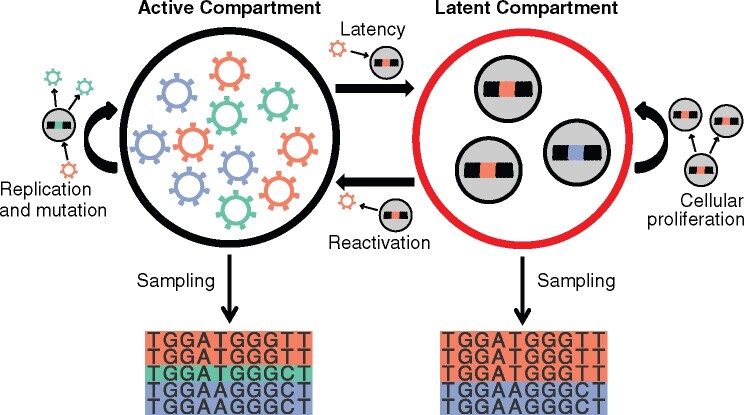
Diagram of simulation model. The black outlined circle represents the active compartments and the red outlined circle represents the latent reservoir. Thick black arrows between compartments represent gene flow, curved black arrows denote replication and mutation, while thin black arrows depict sampling. Genomes replicate and mutate in the active compartment and genomes proliferate, but do not mutate, in the latent compartment. Genomes move from the active compartment to the latent compartment via latency and genomes move from the latent compartment to the active compartment via reactivation. Alignments are sampled from each compartment at specified time points.

With our modified SANTA-SIM, we created a simulation model of HIV-1 evolution within host including the HIV-1 persistent reservoir. Each step of the model corresponds to 2.6 days, which is the approximate duration of the HIV-1 life cycle (Perelson et al. 1996). This model has two compartments: 1, an active compartment representing HIV-1 viruses in blood and plasma that replicate and evolve according to a mutation rate of 9.3 × 10^−5^ mutations per nucleotide site per generation (Cuevas et al. 2015; Perelson et al. 1996) and rate bias matrix:

$$\begin{array}{ll} & \begin{array}{llll} \;A & \;C & \;G & \;T \end{array}\\ \begin{array}{l} A\\ C\\ G\\ T \end{array} & \left(\begin{array}{llll} - & 0.42 & 2.49 & 0.29\\ 1.73 & 1 & 0.23 & 4.73\\ 6.99 & 0.20 & - & 0.60\\ 1.02 & 2.5+ & 0.88 & - \end{array}\right) \end{array}$$

from Jariani et al. (2019) and 2, a latent compartment representing HIV-1 proviruses in the HIV-1 reservoir that cannot evolve but are able to clonally reproduce without mutation at a slow rate simulating homeostatic proliferation (Fig. 1).

The active compartment begins the simulation with 10 genomes that are copies of full-length (9,719 bases) ancestral HIV-1 type B strain HX-B2 (GenBank accession: K03455) except 1, that nucleotide position 9,167 is a guanine (G) instead of an adenine (A) to change the premature stop codon in HX-B2’s *nef* into tryptophan (W) and 2, the nucleotide at position 6,063 is a thymine (T) instead of a cytosine (C) to change the threonine (T) into a start codon. Genomes in the active compartment are subject to selection due to CD4 and HLA-I down-modulation in *nef* based on (Barton et al. 2019). Specifically, for each codon polymorphism investigated we set the fitness value at that site to the observed replication capacity, amino acids seen in NLK-43 and HX-B2 at those sites were given a fitness value of 1 (unless it was the same as the amino acid investigated) and all other amino acids at those sites were given an arbitrary fitness value of 0.001. We also enforced open reading frames in the genome by assigning zero fitness to stop codons in the *gag*, *pol*, *vif*, *vpu*, *env*, and *nef* and a relative fitness of 0.001 to non-start codons at the first positions of *gag*, *vif*, *vpu*, *env*, and *nef*. *tat*, *rev*, and *vpr* were not included for simplicity as *tat* and *rev* are split over multiple reading frame and position in the HIV-1 genome and *vpr* has an insertion in HX-B2. The active compartment follows a logistic population growth model with a growth rate of 50 replicates per generation (derived from Bui, Mellors, and Cillo 2016) and a carrying capacity of 10^5^ viruses (a typical viral load in 1 mL of plasma).

The latent compartment has neither mutation nor a fitness landscape driving its evolution; however, the latent compartment undergoes clonal replication via a birth death population growth model with a birth rate of 0.003 splits per generation (derived from Macallan et al. (2003) and a death rate of 0.0056 deaths per generation (Rong and Perelson 2009), simulating homeostatic proliferation and clonal expansion of infected cells.

The genomes are able to freely migrate between compartments based on a transfer rate matrix where the rate to move from the active compartment to the latent compartment is 2.6 × 10^−3^ genomes per generation (Rong and Perelson 2009) and the rate to move from the latent compartment to the active compartment is 1.08 × 10^−3^ genomes per generation. The rate that genomes in the latent compartment reactivate and enter the active compartment was chosen to make the half-life of the HIV-1 reservoir 70 weeks, based on the 3-month half-life in (Strain et al. 2005).

The simulation contains two epochs. In the first epoch, the simulation proceeds as described above with the active compartment replicating and mutating representing an active HIV-1 infection. In the second epoch, the fitness of the active compartment is always set to zero, resulting in the depletion of viruses in the active compartment; this represents the patient on cART. Full genome alignments of ten genomes are sampled from the active compartment longitudinally every year during the first epoch. Full genomes alignments of ten genomes are sampled longitudinally from the latent compartment longitudinally every two years during the second epoch. The specification XML file for our simulation is included in the Supplementary Materials as an example.

## 4. Simulation results

**Figure 2.**
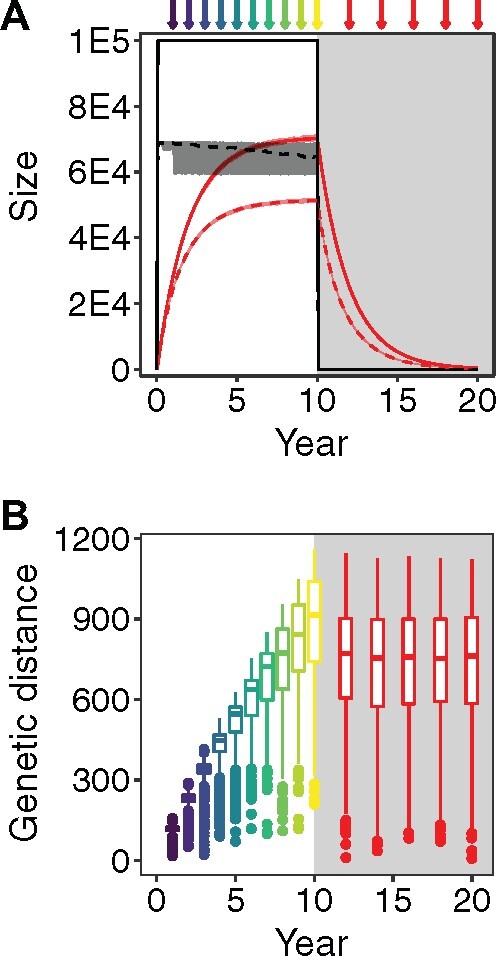
Simulation results. (A) Solid lines represent the mean number of genomes in each compartment over time among the 100 simulated data sets (active = black, latent = red) with surrounding shading representing the range of the number of genomes across all simulations. Dashed lines represent the mean number of lineages in each compartment over time among the 100 simulated data sets (active = black, latent = red) with surrounding shading representing the range of the number of lineages across all simulations. Arrows represent alignment sampling events (active = purple shades earlier and yellow shades later, latent = red). Light grey shading indicates therapy. (B) Box plots of the genetic distances from HX-B2 (nucleotide substitutions) of the sampled full-length (9,719 bases) genomes in each of the 100 simulated data sets (active = purple shades earlier and yellow shades later, latent = red). Light grey shading indicates therapy (*n* = 1,000 per box plot). Grey shading indicates therapy.

We created 100 simulated data sets using our model in our modified SANTA-SIM. Each data set comprised 100 full-length genomes sampled from the active compartment prior to therapy and 50 full-length genomes sampled from the latent compartment after therapy. Figure 2 shows the number of genomes and the genetic divergence over time in the two compartments. The latent compartment achieved its maximum size (median 70,254 [interquartile range (IQR) 70,036–70,550] genomes) at the start of therapy and then decayed. The overall genetic distance of the genomes in the active compartment increased over time and the latent reservoir was mostly comprised genetically distant genomes, but with genetic distances observed throughout the span of active genome distances.

To investigate how our choice of parameters affected the results of the simulation, we varied the parameters of our model and ran simulations with these varied parameters (Supplementary Table S1). In addition to adjusting sampling frequency, sampling depth, reactivation rate, latency rate, latent growth rate, latent death rate, active growth rate, and mutation rate, we also removed and added fitness factors and incorporated recombination and indels. Furthermore, we created a simulation in which we sampled from the latent compartment during active infection. Finally, though we chose to begin our simulations with the ancestral HIV-1 genome HXB2, it is not required to start with that genome. We chose HXB2 because of its historical importance and its use as a standard for HIV-1 genome positioning. Thus, we also created a model starting with a full-length HIV-1 subtype C genome from Malawi (GenBank Accession KC156214) (Parrish et al., 2013). This model did not have selection due to CD4 and HLA-I down-modulation in *nef* because the parameters for the fitness function are specific to subtype B, but the subtype C model contains coding regions adjusted for its genome. Parameters that we chose not to vary include the active population carrying capacity, the substitution rate biases, infection and simulation duration, and specific values for the fitness functions. However, all these parameters can be readily adjusted in the model specification XML file.

Overall, the population size and genetic distance distributions were similar to the main 100 simulated data sets. Plots akin to the figures of the main article are shown in Supplementary Figures S3–S24 together with plots for a representative simulated data set (Supplementary Figure S2) from the main 100 simulated data sets; this representative data set had the smallest difference of mean and SD of sampled integration dates of genomes from the latent compartment from the median of the mean and median of the SD, respectively, of integration dates of genomes from the latent compartment among all 100 simulated data sets. In the simulation where the active growth rate was decreased, the active population did not immediately reach its carrying capacity as it did in all of the other simulations. The population size of the latent compartment exceeded the size of the active compartment in simulations where latency rate was increased, latent growth rate was increased or latent death rate was decreased. We recorded the number of lineages in each compartment over time where a lineage corresponds to a group of genomes who share a common ancestor and have no mutation between them or their ancestor. Overall, the proportion of lineages to the number of genomes was consistent across the simulated data sets. However, the simulation with a higher mutation rate had nearly the same number of lineages as genomes in the active compartment and the simulation with a lower mutation rate had few lineages in the active compartment.

## 5. Comparison to empirical data

Next, we compared our simulated data with data derived from an actual HIV-1 infected individual. We curated HIV-1 *nef* sequences sampled from an HIV-1-infected individual (first presented by Jones et al. 2018). HIV-1 *nef* RNA sequences were collected longitudinally over 14 time points from plasma in the absence of therapy and HIV-1 *nef* DNA sequences were collected from peripheral blood mononuclear cells (PBMCs) from two time points while the individual was on suppressive cART. These sequences are available on GenBank with the following accession numbers: MG822918, MG822919, MG822923-MG822933, MG822935-MG822997, MG822999-MG823015, and MG823144-MG823170. More details on sample collection and sequencing can be found in Jones et al. (2018).

To compare the simulated and empirical data, we constructed rooted maximum likelihood (ML) phylogenies. First, we clipped the simulated full-length genomes to the *nef* region and then we removed duplicated *nef* sequences keeping the earliest sampled sequence. We inferred ML phylogenies from the simulated *nef* sequences and the empirical *nef* sequences using RAxML (Stamatakis 2014), creating one within host phylogeny per data set. Finally, we rooted each phylogeny using the rtt function in the R package ape (Paradis and Schliep 2019), to maximize the correlation between the root to tip distances and collection dates of the sequences from the active compartment.

**Figure 3.**
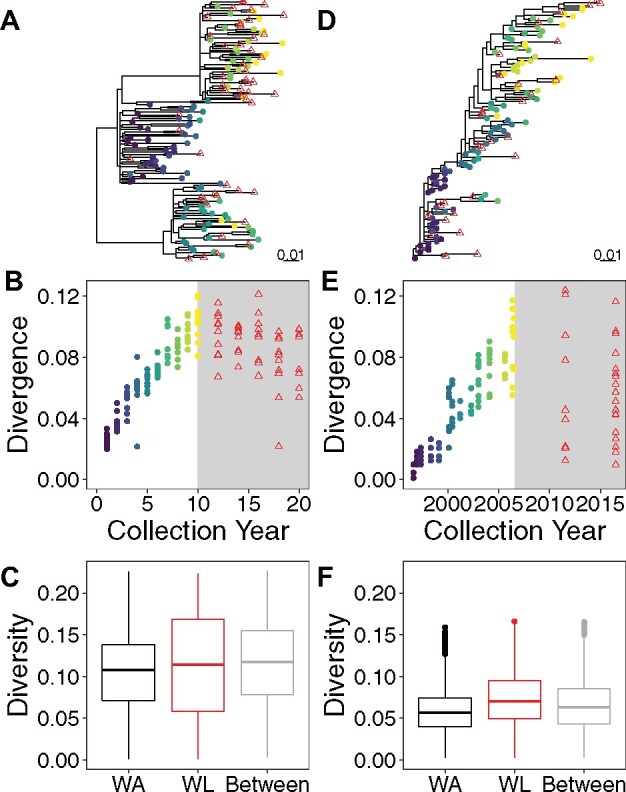
Phylogenetic diversity and divergence of simulated and empirical data. (A–C) A simulated data set chosen from the 100 simulated data sets such that the mean and SD of the sampled reservoir sequence integration dates have minimal deviation from the medians of the mean and SD of the sampled reservoir sequence integration dates among each of the 100 simulated data sets. (D–F) Empirical data derived from an HIV-01-infected individual. (A) Rooted ML phylogeny inferred from *nef* sequences of simulated data. Reservoir sequences appear as red triangles and active sequences appear as circles coloured by collection year (with purple shades earlier and yellow shades later). (B) Distance from the root of the phylogeny to each sequence (in nucleotide substitutions per site) versus collection time in simulated data. Reservoir sequences appear as red triangles and active sequences appear as circles coloured by collection year (with purple shades earlier and yellow shades later). Light grey shading indicates a period of suppressive therapy. (C) Tip-to-tip distances (in nucleotide substitutions per site) between active sequences (WA), between latent sequences (WL) and from active sequences to latent sequences (between) in simulated data (*n* = 4,851; 1,176; 4,851, respectively). (D) Rooted ML phylogeny inferred from *nef* sequences of empirical data. Reservoir sequences appear as red triangles and active sequences appear as circles colored by collection year (with purple shades earlier and yellow shades later). (E) Distance from the root of the phylogeny to each sequence (in nucleotide substitutions per site) versus collection time in empirical data. Reservoir sequences appear as red triangles and active sequences appear as circles colored by collection year (with purple shades earlier and yellow shades later). Light grey shading indicates a period of suppressive therapy. (F) Tip-to-tip distances (in nucleotide substitutions per site) between active sequences (WA), between latent sequences (WL) and from active sequences to latent sequences (between) in empirical data (*n* = 4,278; 406; 2,697, respectively).

**Figure 4.**
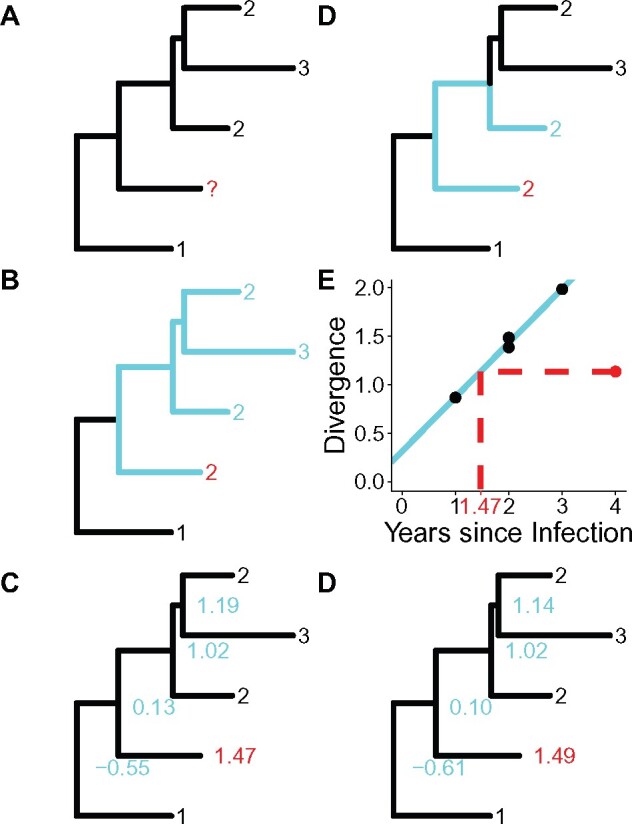
Example illustrations of date estimation methods. (A) An example phylogenetic tree used in each subfigure example. The black numbers represent the collection dates of active sequences. The red question mark represents a latent sequence (query sequence) for which we will estimate the integration date. (B) CS: we find the closest sequence (light blue path) to our query sequence and assign the date of the closest sequence (light blue number) to the query sequence (red number). (C) CD: we find the smallest subtree containing the query sequence and at least one active sequence (light blue subtree). We then take the mode of the dates of the active sequences in the subtree (light blue numbers) and assign it as the query sequence date (red number). (D) LR: we find the linear regression of the collection dates versus the divergence of the active sequences (solid light blue line). Using the divergence of the query, we compute the date (dotted red line) using the linear regression. (E) LS: we assign dates to the internal nodes (light blue numbers) and the query sequences (red number) so as to minimize the divergence between the evolutionary rate of the branches and the difference in the times at the start and end of the branches. (F) ML: we assign dates to the internal nodes (light blue numbers) and the query sequences (red number) to maximize the likelihood of the time scaled tree.

Phylogenies inferred from the simulated data displayed substantial variation in topology (Fig. 4A and Supplementary Figure S25). In Fig. 3, we compare a representative simulated data set with the empirical data described above. The latent sequences sampled from the representative simulated data set displayed higher relative divergence than the empirical data set suggestive of later seeding of the sampled sequences. This is consistent with other empirical HIV reservoir data sets (Brodin et al. 2016; Abrahams et al. 2019). The diversity of the sequences was also higher in the simulated data set. However, the diversity within and between compartments was similar in both types of data set. The divergence over time of the active sequences of the simulated and empirical data were similar. The empirical data had an evolutionary rate of 8.08 × 10^−3^ nucleotide substitutions per site per year and the simulated data sets had a median evolutionary rate of 9.80 × 10^−3^ [IQR 8.02–10.9 × 10^−3^] nucleotide substitutions per site per year. Evolutionary rate was estimated via linear regression between the root to tip distances and collection dates of the active sequences.

The phylogenies of the simulated data sets with varied parameters were within the scope of the phylogenies from the main 100 simulated data sets (Supplementary Figures S3E–S24E). Except for the data sets with different mutation rates, the evolutionary rates of the variable data sets were similar to the evolutionary rates observed in the main simulations (Supplementary Table S1).

## 6. Comparing proviral integration date estimation methods

In HIV-1 persistence research, the timing of integration and duration of persistence of the HIV-1 reservoir is hotly contested. Three studies have attempted to resolve this debate (Brodin et al. 2016; Jones et al. 2018; Abrahams et al. 2019), each using a different method to estimate dates of integration in the HIV-1 reservoir. As a demonstration of our simulation model’s capabilities, we chose to apply a series of five date estimation methods, all of which were phylogenetically based, to the simulated data sets generated by our model using the phylogenies inferred from unique *nef* sequences described in the previous section. Next, we assessed the accuracy of each method to investigate, which method is most appropriate for estimating the integration dates of latent HIV-1 proviruses.

The first method is Closest Sequence (CS), which entails assigning the date of a reservoir sequence based on the date of the phylogenetically closest active sequence. This method was one of three methods used by Abrahams et al. (2019) to infer the integration dates of reservoir sequences. Our second method is Clade (CD), which assigns the date of the reservoir sequence based on the dates of active sequences in the smallest subtree containing the reservoir sequence. The third method is Linear Regression (LR), which involves training a linear regression with the active sequences and then estimating the integration dates of the reservoir sequences using the regression. This method was used by Jones et al. (2018) and Brooks et al. (2020) to infer the timing of integration of proviral sequences. The penultimate method is Least Squares (LS), which aims to mimimize the variance between the dates and the branch lengths of the tree. This method employs the software: LSD (To et al. 2016); originally designed for estimating divergence dates, the latest version of LSD includes estimating sequence ages. The final method is ML, which selects dates to maximize a likelihood function. For this study we used a modified version of node.dating (Jones and Poon 2016), which like LSD can also estimate sequence ages in the latest version. More detailed methods can be found in the last section of the text and diagrams illustrating the methods can be found in Fig. 4.

## 7. Simulated data

**Figure 5.**
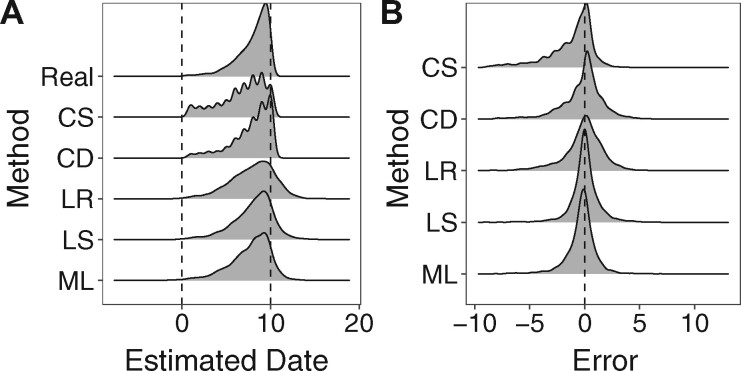
Date estimation and error. (A) Density plot of the integration dates of all 100 simulated data sets (Real) and density plots of the estimated integration dates of all 100 simulated data sets using each method (*n* = 4,196 per density plot). (B) Density plots of the error of estimating the integration each reservoir genome in all 100 simulated data sets using each method (*n* = 4,196 per density plot).

We applied the five date estimation methods to estimate the integration dates of the unique reservoir genomes to each of our 100 simulated data sets. The distributions of real and estimated integration dates are shown in Fig. 5A and error metrics are shown in Fig. 5B and Table 1. On these data, the Least Squares (LS) method was the most accurate with the lowest root mean square error, highest concordance (Table 1) and lower absolute errors (Friedman and all pairwise *t* tests: *P* < 0.01). The only method whose error had significant skewness was the CS method, which had a negative skewness (−1.31). Together with this method’s highly negative median error of −0.596 years indicates a tendency for this method to estimate older dates. The methods overall had negative skewness but were all greater than −1. The negative skewness of the methods may be a result of the distribution of the actual integration dates being skewed to younger dates and not an actual preference for estimating older dates.

**Table 1.** Date estimation method performance of all estimated dates in all 100 simulated data sets (*n* = 4,196).

Method	RMSE (years)	Concordance	Median Error (years)	Skewness
CS	1.58	0.498	−0.596	−1.31
CD	1.32	0.556	0.114	−0.89
LR	1.31	0.645	0.067	−0.55
LS	0.85	0.797	0.014	−0.51
ML	0.92	0.768	−0.184	−0.49

There are many reasons why the LS method may outperform the other methods. Since the LS method treats time as a continuous variable, it is not restricted to the sampled time points for the estimated dates like in the CS and CD methods. The LR method assumes that the data follow a strict molecular clock, which is not held over longer periods of time during within-host HIV-1 infection (Shankarappa et al. 1999), and the LR method is naive to the topology of the phylogeny. The LS and ML methods instead allow variable evolutionary rates over the edges of the phylogeny. Finally, the LS method estimates the overall evolutionary rate while running whereas the ML method uses a fixed overall evolutionary rate that is precomputed by linear regression. The flexibility of the LS method coupled with its statistical framework is probably why it achieves lower error over the other methods.

Finally, we investigated how varying the model parameters would affect the date estimation performance. The actual integration dates of the simulations all skew towards the start of therapy (Fig. 5A and Supplementary Figures S3F–S24F), consistent with previous studies (Brodin et al. 2016; Abrahams et al. 2019). In Supplementary Table S1, we recorded the method with the highest concordance for each data set. Though every method was represented, the LS method performed the best in more data sets. Notably, changing the sampling frequency or depth did not change the preferred method from LS. Also, the CS and CD methods, which heavily rely on sampled time points, perform poorly when the sample frequency and depth are decreased. Comparing the error distributions of the methods across data sets (Supplementary Figures S3G–S24G) reveals that the LS method in general has a tight symmetrical error distribution. Notably, when we added indels (Supplementary Figure S22) or even recombination (Supplementary Figure S23), the LS method still performed well with concordances between estimated and real dates of 0.794 and 0.983, respectively, though the CS method performed best for the simulation with recombination with a concordance between estimated and real dates of 0.989. For the simulation with recombination, we retained all of the sequences regardless of whether they were recombinants or not. This is not advised in general, since recombination violates the hypothesis of phylogenetics that each lineage has only one parent.

To compare the effect of sequence length on the date estimation methods, we considered alignments with different numbers of bases. First, we clipped the simulated data shown in Fig. 3 and Supplementary Figure S2 from nucleotide positions 9,163 to 9,225 (*nef* codons: 123–143) to generate an alignment 63 nucleotide bases long for one data set. For comparison, *nef* is 618 bases long. We chose this region because it contained the most (four) amino positions under selection, according to our fitness model, than any other 63 nucleotide base long alignment. We used the full genome of the same data for a second data set. We removed duplicate sequences and inferred phylogenies from these alignments as previously described and performed the five date estimation methods. The results are shown in Supplementary Figures S26 and S27. The LR method had the highest concordance between estimated and actual dates (0.571) for the 63 base long alignment and the ML method had the highest concordance between estimated and actual dates (0.844) for the full genome alignment. The LS method did not perform much worse than the ML method in the full genome alignment with a concordance between estimated and actual dates of 0.834; however, the LS method performed poorly on the 63 base long alignment with a concordance between estimated and actual dates of 0.413. For context, the concordance between estimated and actual dates of the LS method on the original data set with a full *nef* alignment shown in Supplementary Figure S2 was 0.783. Overall, the methods perform better on longer alignments. The error distributions of all methods were worse for the 63 base long alignment (see Supplementary Figure S26F) with lots of under and over estimation. In the LS method, there was a sequence that dated to 6.47 years after therapy initiation (see the fifth distribution from the top in Supplementary Figure S26E). Note, it is not possible for genomes to enter the reservoir during therapy in our simulations. Estimating dates later than sampling time is a possibility for the LS method and also the LR and ML methods. This phenomenon has been observed in empirical data using linear regression (Jones et al. 2018); however, the 95% confidence intervals of the estimates contained the sampling dates. The CS and CD methods do not have this drawback because they can only give estimated dates that are from sampled time points of active sequences, which is itself a drawback.

## 8. Empirical data

Since simulations do not necessarily capture the entire complexity of the real world, we sought to compare the date estimation methods on our empirical data. We applied the five date estimation methods on the phylogeny derived from the patient data introduced in the “Comparison to empirical data” section to infer the integration dates of the proviral sequences. The date estimation methods for the empirical data were performed in the same manner as for the simulated data with the plasma-derived RNA sequences treated as coming from the active compartment and the PBMC-derived DNA sequences treated as coming from the latent compartment. Although we cannot be certain about the actual integration dates in the empirical data, we can compare the estimates from each method (Supplementary Table S2 and Supplementary Figure S28). In terms of root mean squared deviation (RMSD) and concordance between estimates, the LS and ML were most similar. This is consistent with our findings in simulated data, where these methods outperformed the other methods. The most extreme difference between methods was between the CD and LS methods, which had an RMSD of 2.60 years and a concordance of 0.570. Overall, this is not a significant difference in the estimates; the Pearson correlation coefficient between the estimates for the CD and LS methods was 0.573 with a *P*-value <0.01. These results agree with the results from our *in silico* data, where the LS method performed the most accurately.

## 9. Conclusions

Here, we present an extension of SANTA-SIM that enables the simulation of virus evolution in multiple compartments. Within this software we created simulated data sets of within host HIV-1 evolution including the HIV-1 reservoir. Our simulated data sets moderately resembled empirical data. Next, we utilized the simulated data sets to compare five date estimation methods to recover the estimated integration dates of reservoir genomes. Overall, we found that the LS method implementing LSD (To et al., 2016) yielded the most accurate estimates of the real integration dates.

Our model of HIV-1 evolution does not capture all of HIV-1’s evolutionary characteristics. Most notably, it does not incorporate recombination nor insertions and deletions in the genome, all of which are common in HIV-1 (Clavel et al. 1989; Vartanian et al. 1991; Wood et al. 2009). The decay of the HIV-1 persistent reservoir is not strictly exponential as our model assumes, but instead its half-life lengthens over time (Strain et al. 2005). We also recognize that CD4 and HLA-I down-modulation in *nef* are far from the only evolutionary pressures faced by HIV-1. For example: cytokine, chemokine, SERINC3 and SERINC5 regulation (Grant and Larijani 2017; Usami, Wu, and Göttlinger 2015) and co-receptor tropism (Berger, Murphy, and Farber 1999; Delobel et al. 2005) all affect HIV-1 fitness within host. Our simulation assumes that cART offers a completely inhospitable environment for active HIV-1 with a fitness function of zero; however, HIV-1 drug resistance resulting from point mutations can and does occur resulting in detectable viral loads and viral evolution (Shafer 1998; Ledergerber et al. 1999; Wang et al. 2011). PBMCs in the blood with transcriptionally latent HIV-1 provirus do not constitute the entirety of HIV-1 in an individual on cART. HIV-1 can also persist in anatomical reservoirs including but not limited to: lymphoid tissue (Finzi et al. 1999), cerebrospinal fluid (Rose et al. 2016; Oliveira et al. 2017) and male and female reproductive organs (Bull et al. 2009; Miller et al. 2019); that may contribute to viral rebound after cessation of cART (Rothenberger et al. 2015; De Scheerder et al. 2019). These features are all planned for later iterations of our model.

The date estimation methods that we tested are not meant to be exhaustive. Brodin et al. (2016) used next-generation sequencing to create genetic signatures for each time point and matched reservoir sequences based on how well they fit the signature. Abrahams et al. (2019), in addition to the CS tested in this paper, used phylogenetic placement and a variation of the CD method to estimate integration dates of reservoir sequences. Additionally, Bayesian methods most notably with BEAST (Shapiro et al. 2011; Suchard et al. 2018; Bouckaert et al. 2019; ) can be employed to estimate unknown sequence ages. In this study, we restricted the scope of our methods to those that are limited to a fixed tree topology.

In addition to HIV-1, our modified SANTA-SIM with multiple compartment functionality could be applied to other viruses. For example, multiple compartments could be specified for cases of zoonosis in viral epidemics (Dudas et al. 2018; Glennon et al. 2019). This would allow modelling separate selection and population growth models for human and animal reservoir viral populations.

Our software provides a useful tool for validating phylodynamic methods developed for the HIV-1 reservoir, helping us understand the dynamics of the HIV-1 reservoir, thus bringing us closer to a durable HIV-1 cure.

## 10. Proviral integration date estimation methodology

The first step in the pipeline of each method was to clip the genomes to one gene (*nef*) using R v3.6.2 (R Core Team 2020) with the R package seqinr (Charif and Lobry 2007). We removed duplicate sequences retaining the oldest sequence of each set of duplicate sequences using a custom R script. Next, we identified the best fitting model using ModelTest-NG v0.1.6 (Darriba et al. 2020) and inferred a ML tree using RAxML v8.2.11 (Stamatakis 2014). Finally, we rooted the trees with root-to-tip regression maximizing the correlation between the sampling dates of the sequences from the active compartment and their divergence from the root with the R package ape (Paradis and Schliep, 2019). Finally, we applied each of the methods detailed in the following sections to the tree. Subsequent statistical analyses and visualization were performed using the R packages: tidyverse (Wickham et al. 2019), ggtree (Yu et al. 2016), and treeio (Wang et al. 2020).

### 10.1 Closest sequence

In the first method, each sequence from the latent compartment is assigned an integration date equal to the sampling date of the closest sequence from the active compartment via patristic distance. In the case of a tie for closest sequence, the mean of the sampling dates is used instead. The CS method was implemented in a custom R script.

### 10.2 Clade

In the second method, first the tree is midpoint rooted. For each query sequence in the latent compartment, the smallest subtree (or clade) that contains the query sequence and at least one sequence from the active compartment is selected. The query sequence is then assigned an integration date equal to the mode of the sampling dates of the sequences from the active compartment that are contained in the selected subtree. The CD method was implemented in a custom R script.

### 10.3 Linear regression

In the third method, a linear regression is inferred comparing the sampling dates versus the patristic distance from the root of the tree of the sequences from the active compartment. The integration dates of the sequences from the latent compartment are inferred from LR using their patristic distance from the root of the tree (Jones et al. 2018).

### 10.4 Least squares

In the penultimate method, the dates of the internal nodes and sequences from the latent component of the tree are selected to minimize the variance between the branch lengths and the difference in time. LSD v0.3.3 (To et al. 2016; To, 2018) was used for the LS dating method.

### 10.5 Maximum likelihood

In the final method, the internal nodes and sequences from the latent compartment are assigned dates to maximize their likelihood. A modified version of node.dating available on GitHub (Jones and Poon 2016; Jones, 2019) was used for the ML method using the evolutionary rate estimated by linear regression as in the LR method.

## Supplementary Material

veaa089_Supplementary_Data
