# Supplementary material for: Simulating within host human immunodeficiency virus 1 genome evolution in the persistent reservoir
Source: Virus Evol. 2020 Nov 23;6(2):veaa089. doi: 10.1093/ve/veaa089 (PMC8132731; doi:10.1093/ve/veaa089)
Supplement: veaa089_Supplementary_Data [file veaa089_supplementary_data.zip › Santa.Sim.Supplemetnary.Materials.pdf]

Supplementary Materials: Simulating within host human immunodeficiency virus 1 genome evolution in the persistent reservoir

Bradley R. Jones, Jeffrey B. Joy

Tables

Supplementary Table 1. Results of simulations with varying parameters.

Simulation	Variable Change	Evolutionary Rate (nucl. subs. / (site year)	Most Concordant Dating Method
**Main**	None	8.42E-03	LS
**Sampling (3)**	Sample 1 year, 5 years, 10 years after infection	9.17E-03	LS
**Sampling (5)**	Sample every 2 years	7.85E-03	LS
**Sampling Size (Low)**	1 genome per sample	1.20E-02	LS
**Sampling Size (High)**	100 genomes per sample	9.70E-03	LS
**Fitness (Neutral)**	Neutral fitness and no coding regions	1.12E-02	ML
**Fitness (HLA)**	Add HLA associated substitutions	1.14E-02	LR
**Reactivation Rate (Low)**	5.4E-4 per gen.	1.28E-02	ML
**Reactivation Rate (High)**	2.16E-3 per gen.	7.47E-03	LS
**Latency Rate (Low)**	1.3E-3 per gen.	1.29E-02	ML
**Latency Rate (High)**	5.2E-3 per gen.	6.53E-03	CD
**Latent Growth Rate (Low)**	2E-3 per gen.	1.21E-02	LS
**Latent Growth Rate (High)**	4.5E-3 per gen	8.44E-03	LS
**Latent Death Rate (Low)**	3.73E-3 per gen.	9.32E-03	LR
**Latent Death Rate (High)**	8.4E-3 per gen.	1.04E-02	LS
**Active Growth Rate (Low)**	5 per gen.	6.46E-03	CS
**Active Growth Rate (High)**	500 per gen.	1.09E-02	ML
**Mutation Rate (Low)**	9.3E-6 nucl. subs./ (site gen.)	7.88E-04	CD
**Mutation Rate (High)**	9.3E-4 nucl. subs. / (site gen.)	9.82E-02	ML
**Recombination**	Add recombination	6.52E-03	CS
**Indel**	Add indels / neutral fitness with no coding regions	1.34E-2	LS
**Early Latent Sampling**	Sample from latent compartment every 2 years	1.31E-2	LS
**Subtype C**	KC156214 starting virus	1.08E-02	LS

CS: Closest Sequence, CD: Clade, LR: Linear Regression, LS: Least Squares, ML: Maximum Likelihood.

Supplementary Table 2. Concordance and root mean squared deviation of date estimation methods using empirical data. (n = 29)

	**Root mean squared deviation (years)**				
**Concordance**	**CS**	1.24	1.86	2.06	1.64
	0.782	**CD**	2.32	2.60	2.00
	0.814	0.694	**LR**	1.29	1.39
	0.740	0.570	0.904	**LS**	0.944
	0.812	0.695	0.900	0.940	**ML**

CS: Closest Sequence, CD: Clade, LR: Linear Regression, LS: Least Squares, ML: Maximum Likelihood

Figures

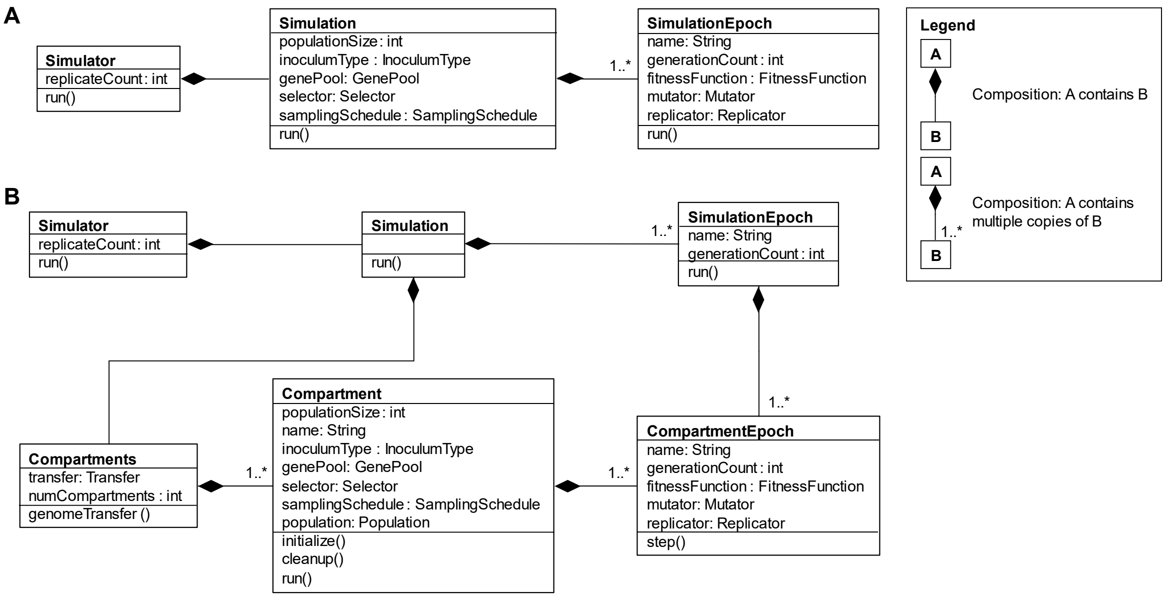

Supplementary Figure S1. SANTA-SIM class diagrams. Reduced class diagrams of (A) SANTA-SIM and (B) our modified SANTA-SIM. These diagrams do not represent the entire class structure of either software. In particular: static members, accessors and mutators or the inheriting classes of the interfaces (InoculumType, GenePool, Selector, SamplingSchedule, FitnessFunction, Mutator, Replicator and Transfer) are not included.

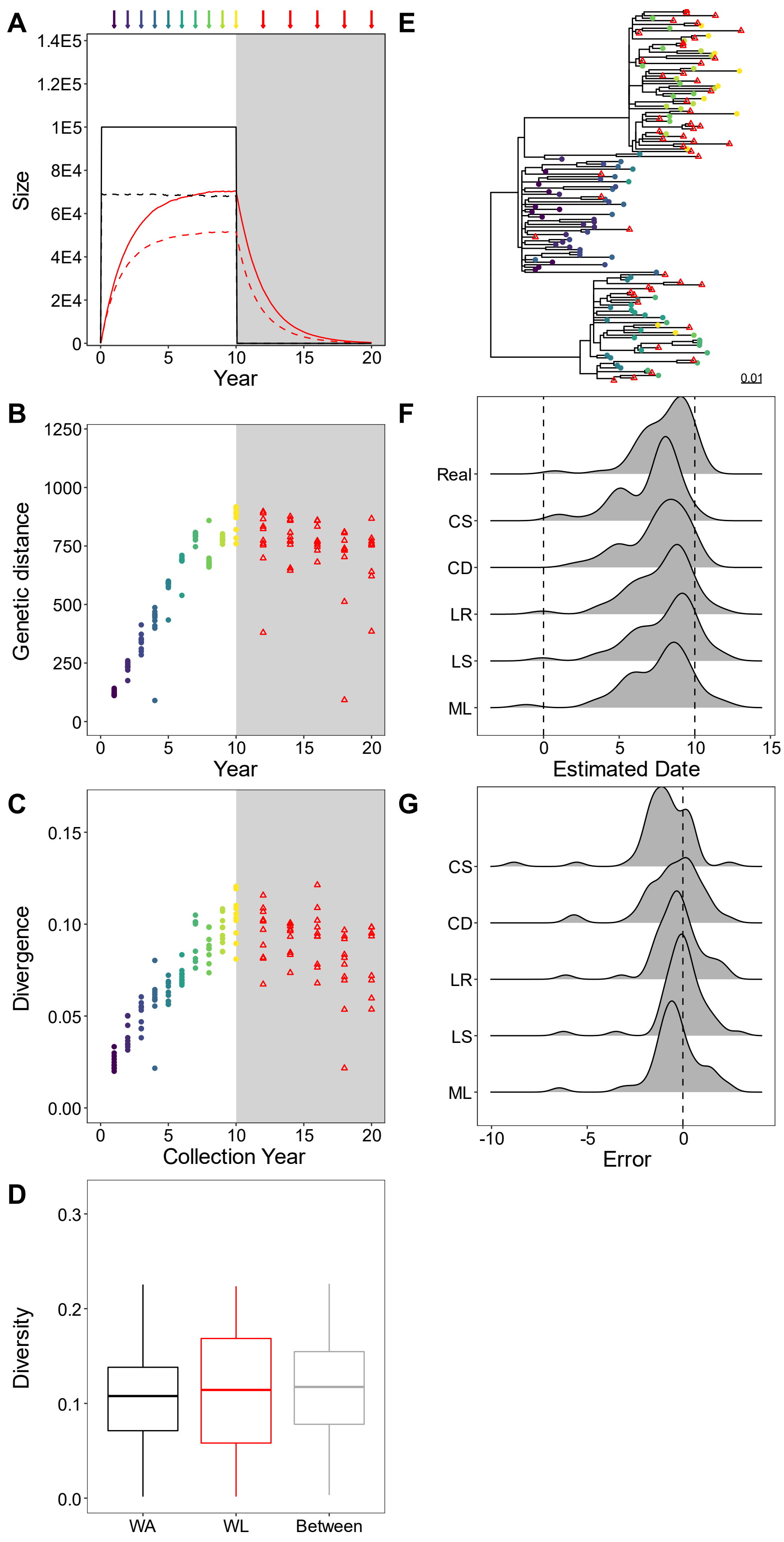
A
Size
Year
E
0.01
B
Genetic distance
Year
F
Real
CS
CD
LR
LS
ML
Estimated Date
C
Divergence
Collection Year
G
CS
CD
LR
LS
ML
Error
D
Diversity
WA
WL
Between

Supplementary Figure S2. Main simulation. Simulated data following the parameters outlined in the main text. This is the same data set shown Figure 3A-C. (A) Solid lines represent the number of genomes in each compartment over time (active = black, latent = red). Dashed lines represent the mean number of lineages in each compartment over time (active = black, latent = red). Arrows represent alignment sampling events (active = purple shades earlier and yellow shades later, latent = red). Grey shading indicates therapy. (B) Genetic distances from HX-B2 (nucleotide substitutions) of the sampled full-length (9719 bases) genomes (active = purple shades earlier and yellow shades later, latent = red). Grey shading indicates therapy. Grey shading indicates therapy. (C) Distance from the root of the phylogeny to each sequence (in nucleotide substitutions per site) versus collection time. Reservoir sequences appear as red triangles and active sequences appear as circles coloured by collection year (with purple shades earlier and yellow shades later). Grey shading indicates a period of suppressive therapy. (D) Tip-to-tip distances (in nucleotide substitutions per site) between active sequences (WA), between latent sequences (WL) and from active sequences to latent sequences (Between). (E) Rooted maximum likelihood phylogeny inferred from *nef* sequences of simulated data. Reservoir sequences appear as red triangles and active sequences appear as circles coloured by collection year (with purple shades earlier and yellow shades later). (F) Density plot of the integration dates (Real) and density plots of the estimated integration dates using each method. (G) Density plots of the error of estimating the integration each reservoir genome. CS: Closest Sequence, CD: Clade, LR: Linear Regression, LS: Least Squares, ML: Maximum Likelihood.

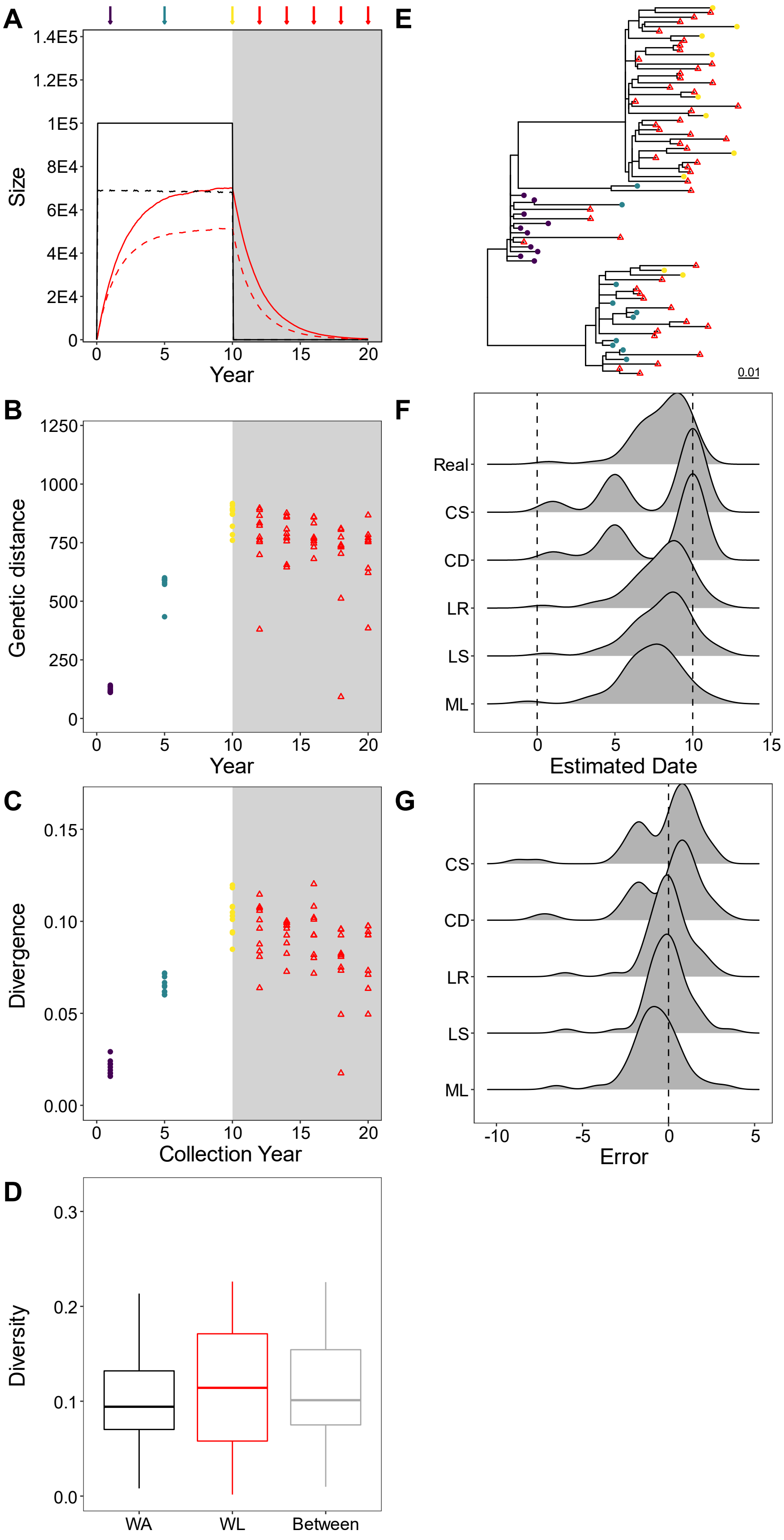

A
Size
1.4E5
1.2E5
1E5
8E4
6E4
4E4
2E4
0
Year
B
Genetic distance
1250
1000
750
500
250
Year
C
Divergence
0.15
0.10
0.05
0.00
Collection Year
D
Diversity
0.3
0.2
0.1
0.0
WA
WL
Between
E
0.01
F
Real
CS
CD
LR
LS
ML
Estimated Date
G
Error

Supplementary Figure S3. Sampling (3). Simulated data with only 3 sampling point from the active compartment at 1 year, 5 years and 10 years after infection. This data set is derived from the data in Supplementary Figure S2 with the active genomes from 2-4 years and 6-9 years removed. (A) Solid lines represent the number of genomes in each compartment over time (active = black, latent = red). Dashed lines represent the mean number of lineages in each compartment over time (active = black, latent = red). Arrows represent alignment sampling events (active = purple shades earlier and yellow shades later, latent = red). Grey shading indicates therapy. (B) Genetic distances from HX-B2 (nucleotide substitutions) of the sampled full-length (9719 bases) genomes (active = purple shades earlier and yellow shades later, latent = red). Grey shading indicates therapy. Grey shading indicates therapy. (C) Distance from the root of the phylogeny to each sequence (in nucleotide substitutions per site) versus collection time. Reservoir sequences appear as red triangles and active sequences appear as circles coloured by collection year (with purple shades earlier and yellow shades later). Grey shading indicates a period of suppressive therapy. (D) Tip-to-tip distances (in nucleotide substitutions per site) between active sequences (WA), between latent sequences (WL) and from active sequences to latent sequences (Between). (E) Rooted maximum likelihood phylogeny inferred from *nef* sequences of simulated data. Reservoir sequences appear as red triangles and active sequences appear as circles coloured by collection year (with purple shades earlier and yellow shades later). (F) Density plot of the integration dates (Real) and density plots of the estimated integration dates using each method. (G) Density plots of the error of estimating the integration each reservoir genome. CS: Closest Sequence, CD: Clade, LR: Linear Regression, LS: Least Squares, ML: Maximum Likelihood.

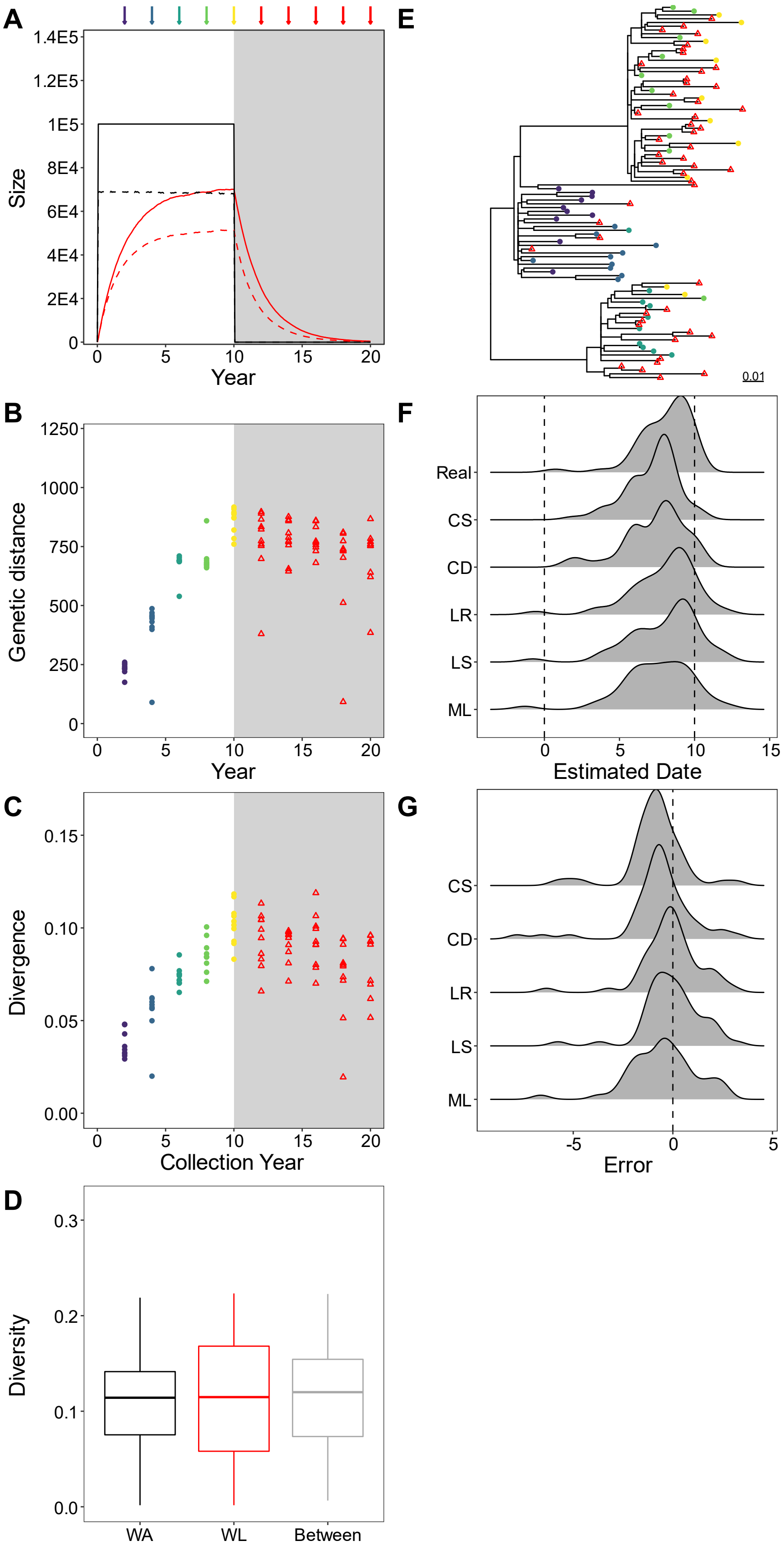

A
Size
1.4E5
1.2E5
1E5
8E4
6E4
4E4
2E4
0
Year
B
Genetic distance
1250
1000
750
500
250
Year
C
Divergence
0.15
0.10
0.05
0.00
Collection Year
D
Diversity
0.3
0.2
0.1
0.0
WA
WL
Between
E
0.01
F
Real
CS
CD
LR
LS
ML
Estimated Date
G
CS
CD
LR
LS
ML
Error

Supplementary Figure S4. Sampling (5). Simulated data with only 5 sampling point from the active compartment every two years after infection. This data set is derived from the data in Supplementary Figure S2 with the active genomes from odd years removed. (A) Solid lines represent the number of genomes in each compartment over time (active = black, latent = red). Dashed lines represent the mean number of lineages in each compartment over time (active = black, latent = red). Arrows represent alignment sampling events (active = purple shades earlier and yellow shades later, latent = red). Grey shading indicates therapy. (B) Genetic distances from HX-B2 (nucleotide substitutions) of the sampled full-length (9719 bases) genomes (active = purple shades earlier and yellow shades later, latent = red). Grey shading indicates therapy. Grey shading indicates therapy. (C) Distance from the root of the phylogeny to each sequence (in nucleotide substitutions per site) versus collection time. Reservoir sequences appear as red triangles and active sequences appear as circles coloured by collection year (with purple shades earlier and yellow shades later). Grey shading indicates a period of suppressive therapy. (D) Tip-to-tip distances (in nucleotide substitutions per site) between active sequences (WA), between latent sequences (WL) and from active sequences to latent sequences (Between). (E) Rooted maximum likelihood phylogeny inferred from *nef* sequences of simulated data. Reservoir sequences appear as red triangles and active sequences appear as circles coloured by collection year (with purple shades earlier and yellow shades later). (F) Density plot of the integration dates (Real) and density plots of the estimated integration dates using each method. (G) Density plots of the error of estimating the integration each reservoir genome. CS: Closest Sequence, CD: Clade, LR: Linear Regression, LS: Least Squares, ML: Maximum Likelihood.

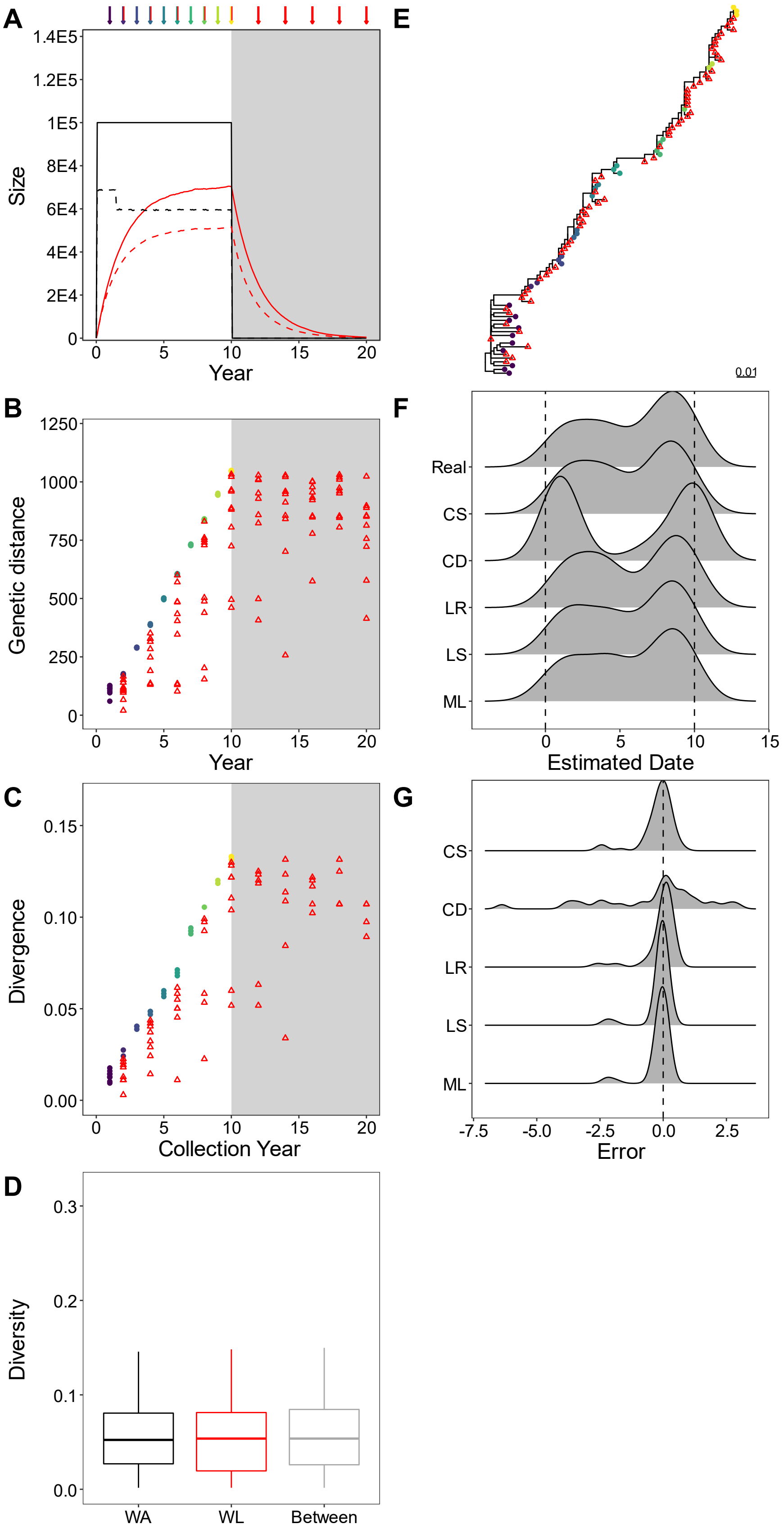

A
Size
1.4E5
1.2E5
1E5
8E4
6E4
4E4
2E4
0
Year
B
Genetic distance
1250
1000
750
500
250
Year
C
Divergence
0.15
0.10
0.05
0.00
Collection Year
D
Diversity
0.3
0.2
0.1
0.0
WA
WL
Between
E
0.01
F
Real
CS
CD
LR
LS
ML
Estimated Date
G
Error
-7.5
-5.0
-2.5
0.0
2.5

Supplementary Figure S5. Early latent sampling. Simulated data set with additional sampling from the latent compartment every 2 years during active infection. (A) Solid lines represent the number of genomes in each compartment over time (active = black, latent = red). Dashed lines represent the mean number of lineages in each compartment over time (active = black, latent = red). Arrows represent alignment sampling events (active = purple shades earlier and yellow shades later, latent = red). Grey shading indicates therapy. (B) Genetic distances from HX-B2 (nucleotide substitutions) of the sampled full-length (9719 bases) genomes (active = purple shades earlier and yellow shades later, latent = red). Grey shading indicates therapy. Grey shading indicates therapy. (C) Distance from the root of the phylogeny to each sequence (in nucleotide substitutions per site) versus collection time. Reservoir sequences appear as red triangles and active sequences appear as circles coloured by collection year (with purple shades earlier and yellow shades later). Grey shading indicates a period of suppressive therapy. (D) Tip-to-tip distances (in nucleotide substitutions per site) between active sequences (WA), between latent sequences (WL) and from active sequences to latent sequences (Between). (E) Rooted maximum likelihood phylogeny inferred from *nef* sequences of simulated data. Reservoir sequences appear as red triangles and active sequences appear as circles coloured by collection year (with purple shades earlier and yellow shades later). (F) Density plot of the integration dates (Real) and density plots of the estimated integration dates using each method. (G) Density plots of the error of estimating the integration each reservoir genome. CS: Closest Sequence, CD: Clade, LR: Linear Regression, LS: Least Squares, ML: Maximum Likelihood.

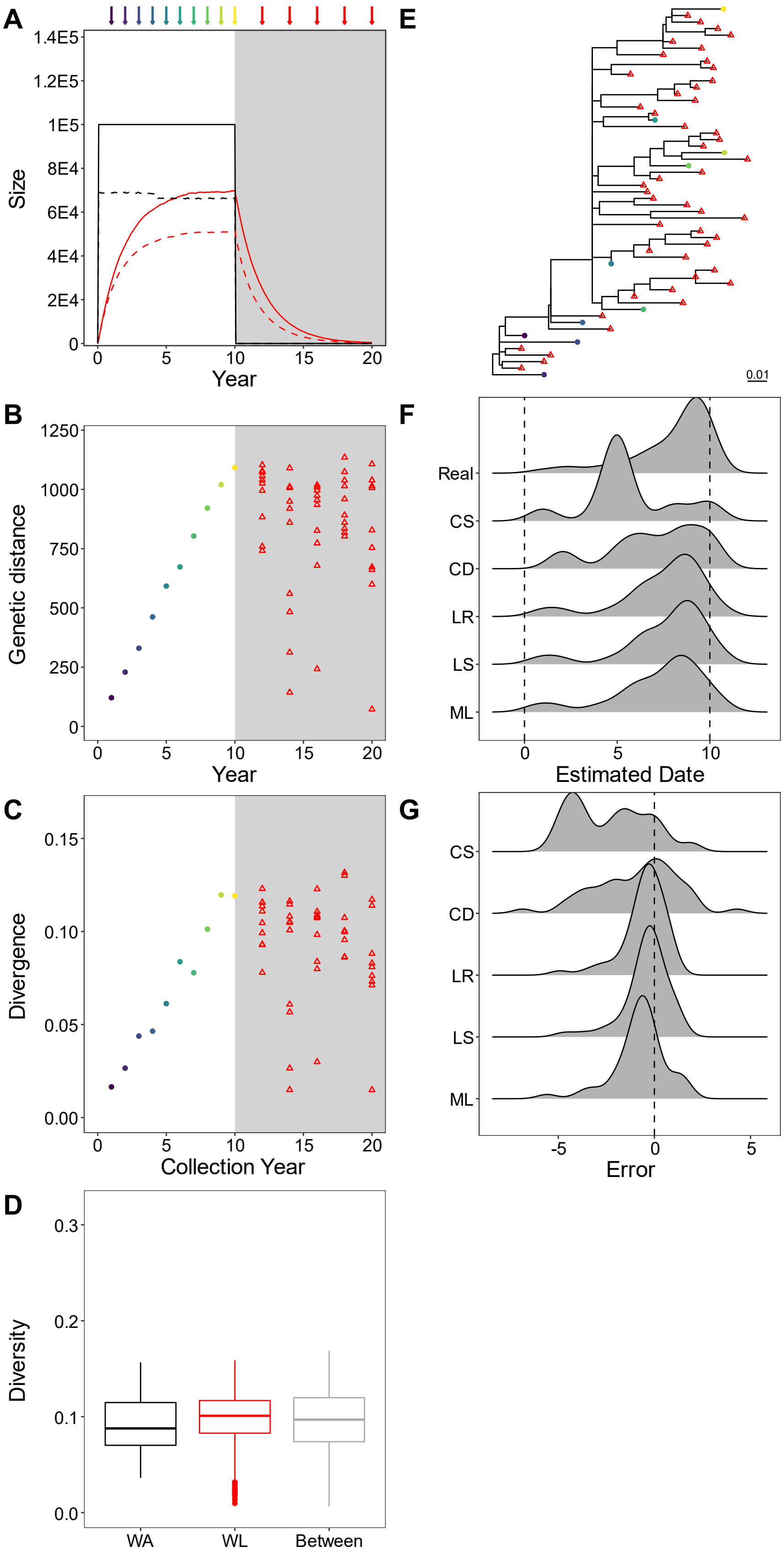
A
B
C
D
E
F
G
Size
Year
Genetic distance
Divergence
Collection Year
Diversity
WA
WL
Between
0.01
Real
CS
CD
LR
LS
ML
Estimated Date
Error

Supplementary Figure S6. Sampling Size (Low). Simulated data set with 1 genome sampled per sampling point from the active compartment. (A) Solid lines represent the number of genomes in each compartment over time (active = black, latent = red). Dashed lines represent the mean number of lineages in each compartment over time (active = black, latent = red). Arrows represent alignment sampling events (active = purple shades earlier and yellow shades later, latent = red). Grey shading indicates therapy. (B) Genetic distances from HX-B2 (nucleotide substitutions) of the sampled full-length (9719 bases) genomes (active = purple shades earlier and yellow shades later, latent = red). Grey shading indicates therapy. Grey shading indicates therapy. (C) Distance from the root of the phylogeny to each sequence (in nucleotide substitutions per site) versus collection time. Reservoir sequences appear as red triangles and active sequences appear as circles coloured by collection year (with purple shades earlier and yellow shades later). Grey shading indicates a period of suppressive therapy. (D) Tip-to-tip distances (in nucleotide substitutions per site) between active sequences (WA), between latent sequences (WL) and from active sequences to latent sequences (Between). (E) Rooted maximum likelihood phylogeny inferred from *nef* sequences of simulated data. Reservoir sequences appear as red triangles and active sequences appear as circles coloured by collection year (with purple shades earlier and yellow shades later). (F) Density plot of the integration dates (Real) and density plots of the estimated integration dates using each method. (G) Density plots of the error of estimating the integration each reservoir genome. CS: Closest Sequence, CD: Clade, LR: Linear Regression, LS: Least Squares, ML: Maximum Likelihood.

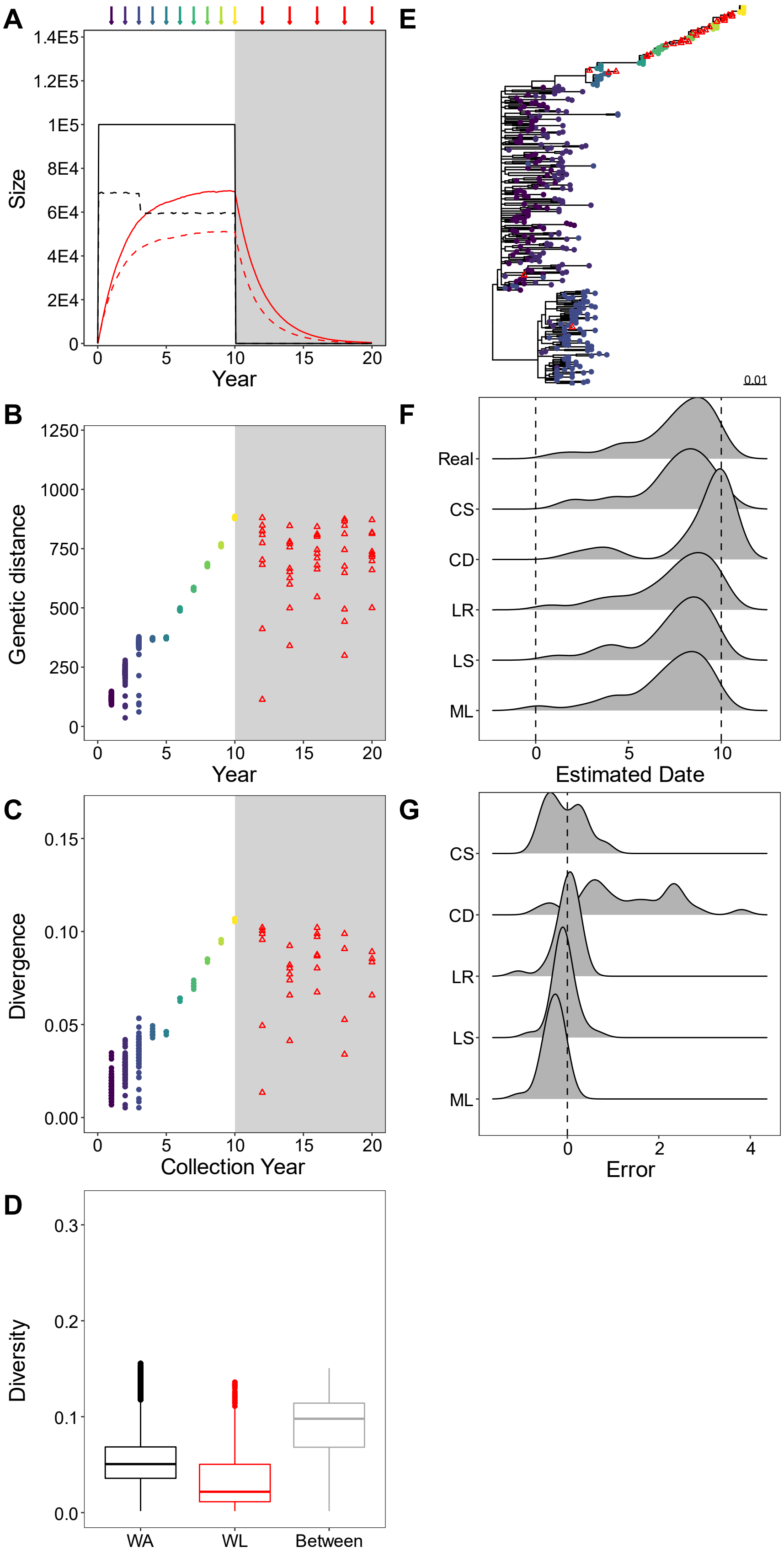

A
Size
1.4E5
1.2E5
1E5
8E4
6E4
4E4
2E4
0
0 5 10 15 20
Year
B
Genetic distance
1250
1000
750
500
250
0
0 5 10 15 20
Year
C
Divergence
0.15
0.10
0.05
0.00
0 5 10 15 20
Collection Year
D
Diversity
0.3
0.2
0.1
0.0
WA
WL
Between
E
0.01
F
Real
CS
CD
LR
LS
ML
0 5 10
Estimated Date
G
CS
CD
LR
LS
ML
0 2 4
Error

Supplementary Figure S7. Sampling Size (High). Simulated data set with 100 genomes sampled per sampling point from the active compartment. (A) Solid lines represent the number of genomes in each compartment over time (active = black, latent = red). Dashed lines represent the mean number of lineages in each compartment over time (active = black, latent = red). Arrows represent alignment sampling events (active = purple shades earlier and yellow shades later, latent = red). Grey shading indicates therapy. (B) Genetic distances from HX-B2 (nucleotide substitutions) of the sampled full-length (9719 bases) genomes (active = purple shades earlier and yellow shades later, latent = red). Grey shading indicates therapy. Grey shading indicates therapy. (C) Distance from the root of the phylogeny to each sequence (in nucleotide substitutions per site) versus collection time. Reservoir sequences appear as red triangles and active sequences appear as circles coloured by collection year (with purple shades earlier and yellow shades later). Grey shading indicates a period of suppressive therapy. (D) Tip-to-tip distances (in nucleotide substitutions per site) between active sequences (WA), between latent sequences (WL) and from active sequences to latent sequences (Between). (E) Rooted maximum likelihood phylogeny inferred from *nef* sequences of simulated data. Reservoir sequences appear as red triangles and active sequences appear as circles coloured by collection year (with purple shades earlier and yellow shades later). (F) Density plot of the integration dates (Real) and density plots of the estimated integration dates using each method. (G) Density plots of the error of estimating the integration each reservoir genome. CS: Closest Sequence, CD: Clade, LR: Linear Regression, LS: Least Squares, ML: Maximum Likelihood.

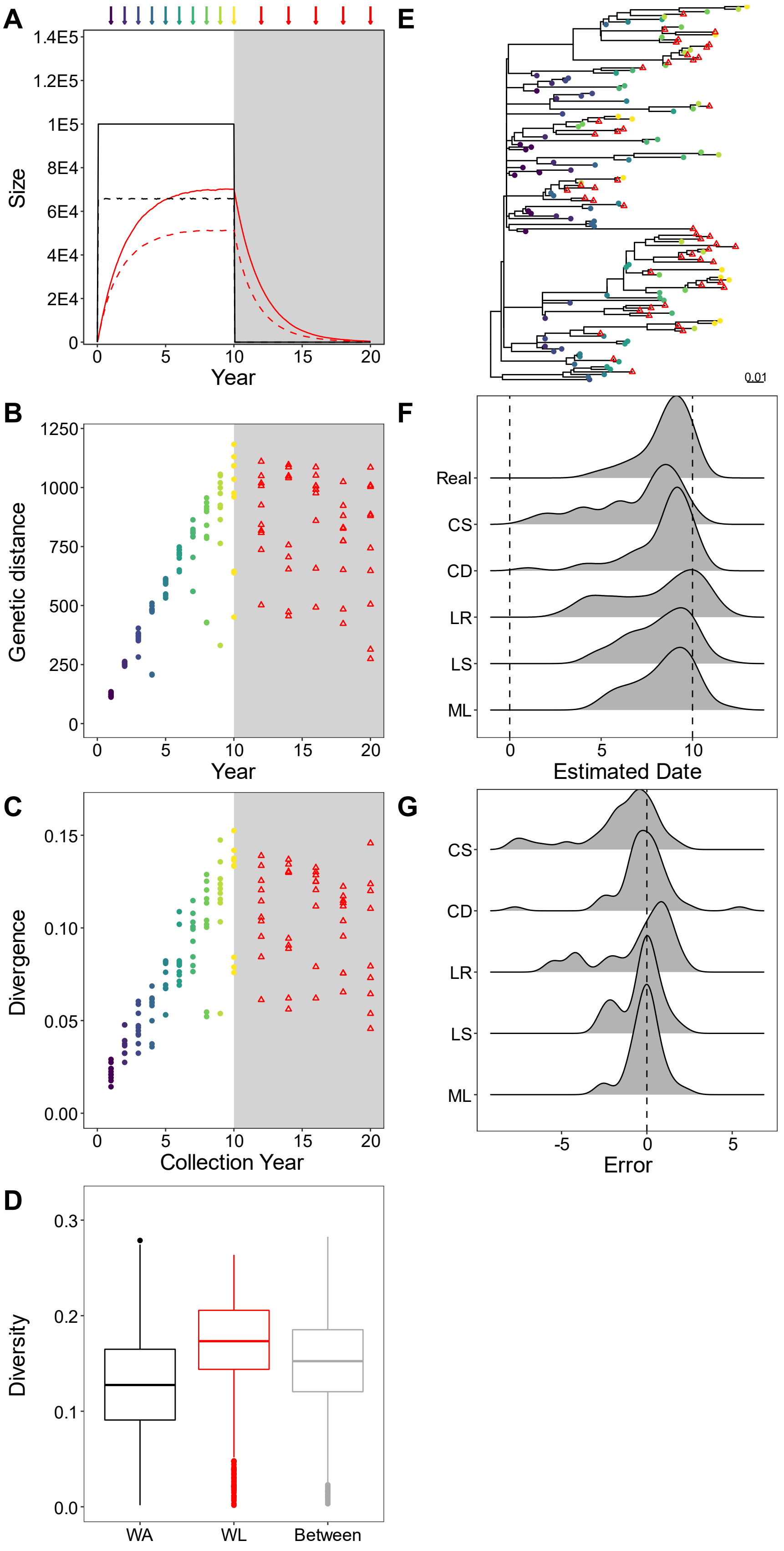

A
B
C
D
E
F
G
Size
Year
Genetic distance
Divergence
Collection Year
Diversity
WA
WL
Between
0.01
Real
CS
CD
LR
LS
ML
Estimated Date
Error

Supplementary Figure S8. Fitness (Neutral). Simulated data set with neutral fitness. Restrictions on coding region (including start and stop codons) also removed. (A) Solid lines represent the number of genomes in each compartment over time (active = black, latent = red). Dashed lines represent the mean number of lineages in each compartment over time (active = black, latent = red). Arrows represent alignment sampling events (active = purple shades earlier and yellow shades later, latent = red). Grey shading indicates therapy. (B) Genetic distances from HX-B2 (nucleotide substitutions) of the sampled full-length (9719 bases) genomes (active = purple shades earlier and yellow shades later, latent = red). Grey shading indicates therapy. Grey shading indicates therapy. (C) Distance from the root of the phylogeny to each sequence (in nucleotide substitutions per site) versus collection time. Reservoir sequences appear as red triangles and active sequences appear as circles coloured by collection year (with purple shades earlier and yellow shades later). Grey shading indicates a period of suppressive therapy. (D) Tip-to-tip distances (in nucleotide substitutions per site) between active sequences (WA), between latent sequences (WL) and from active sequences to latent sequences (Between). (E) Rooted maximum likelihood phylogeny inferred from *nef* sequences of simulated data. Reservoir sequences appear as red triangles and active sequences appear as circles coloured by collection year (with purple shades earlier and yellow shades later). (F) Density plot of the integration dates (Real) and density plots of the estimated integration dates using each method. (G) Density plots of the error of estimating the integration each reservoir genome. CS: Closest Sequence, CD: Clade, LR: Linear Regression, LS: Least Squares, ML: Maximum Likelihood.

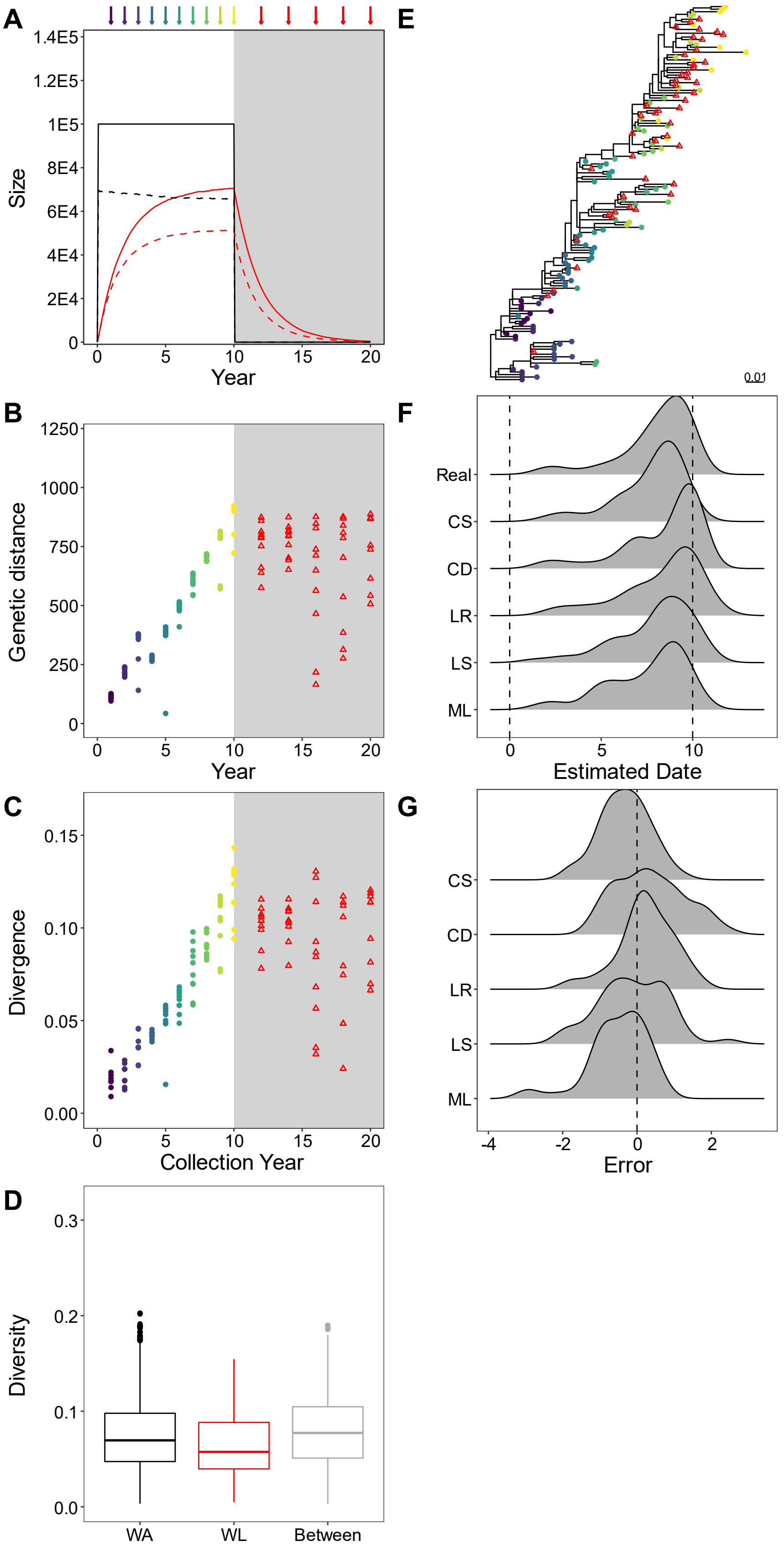

A
Size
1.4E5
1.2E5
1E5
8E4
6E4
4E4
2E4
0
0
5
10
15
20
Year
B
Genetic distance
1250
1000
750
500
250
0
Year
C
Divergence
0.15
0.10
0.05
0.00
Collection Year
D
Diversity
0.3
0.2
0.1
0.0
WA
WL
Between
E
0.01
F
Real
CS
CD
LR
LS
ML
Estimated Date
G
-4
-2
2
Error

Supplementary Figure S9. Fitness (HLA). Simulated data set with additional fitness imparting selection due to host human leukocyte antigen (HLA) A*01 B*08 DRB1*04:01 in *gag*, *pol*, and *nef*. For each substitution found in (Brumme et al., 2009) with a positive or negative association to HLA A1 or B18, if the association was positive and indirect (non-covariation corrected) then substitution was given a fitness value of 1.01, if the association was positive and direct (covariation corrected) then substitution was given a fitness value of 1.02, if the association was negative and indirect then substitution was given a fitness value of 0.99, and if the association was negative and direct then substitution was given a fitness value of 0.98. All other amino acids at those positions were given a fitness value of 1. Selection due to due to CD4 and HLA-I down-modulation in *nef* based on (Barton et al., 2019) and codon restrictions (start and stop codons) were maintained. (A) Solid lines represent the number of genomes in each compartment over time (active = black, latent = red). Dashed lines represent the mean number of lineages in each compartment over time (active = black, latent = red). Arrows represent alignment sampling events (active = purple shades earlier and yellow shades later, latent = red). Grey shading indicates therapy. (B) Genetic distances from HX-B2 (nucleotide substitutions) of the sampled full-length (9719 bases) genomes (active = purple shades earlier and yellow shades later, latent = red). Grey shading indicates therapy. Grey shading indicates therapy. (C) Distance from the root of the phylogeny to each sequence (in nucleotide substitutions per site) versus collection time. Reservoir sequences appear as red triangles and active sequences appear as circles coloured by collection year (with purple shades earlier and yellow shades later). Grey shading indicates a period of suppressive therapy. (D) Tip-to-tip distances (in nucleotide substitutions per site) between active sequences (WA), between latent sequences (WL) and from active sequences to latent sequences (Between). (E) Rooted maximum likelihood phylogeny inferred from *nef* sequences of simulated data. Reservoir sequences appear as red triangles and active sequences appear as circles coloured by collection year (with purple shades earlier and yellow shades later). (F) Density plot of the integration dates (Real) and density plots of the estimated integration dates

using each method. (G) Density plots of the error of estimating the integration each reservoir genome. CS: Closest Sequence, CD: Clade, LR: Linear Regression, LS: Least Squares, ML: Maximum Likelihood.

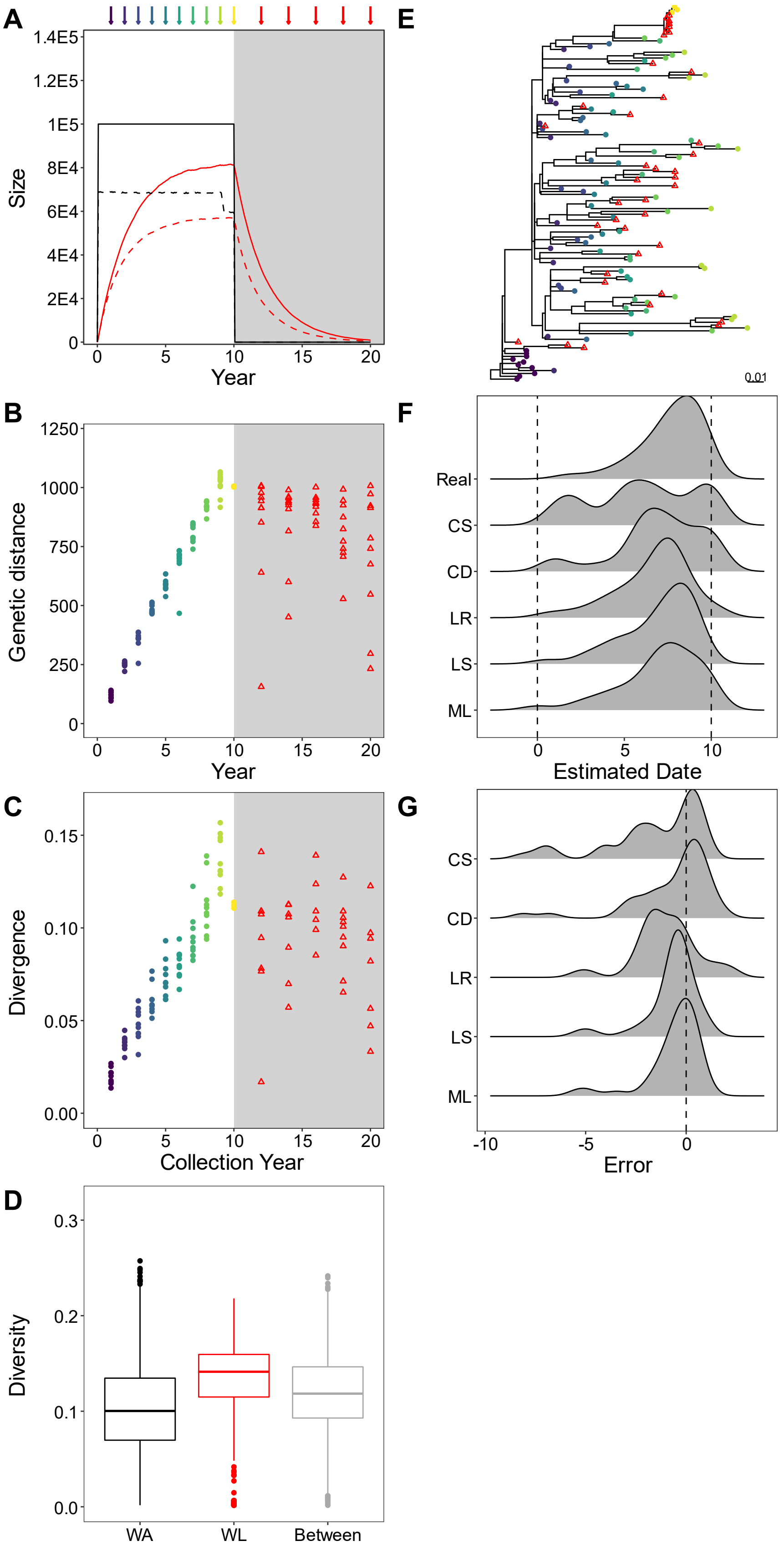
A
Size
1.4E5
1.2E5
1E5
8E4
6E4
4E4
2E4
0
Year
B
Genetic distance
1250
1000
750
500
250
Year
C
Divergence
0.15
0.10
0.05
0.00
Collection Year
D
Diversity
0.3
0.2
0.1
0.0
WA
WL
Between
E
0.01
F
Real
CS
CD
LR
LS
ML
Estimated Date
G
CS
CD
LR
LS
ML
Error
-10
-5
0

Supplementary Figure S10. Reactivation Rate (Low). Simulated data set with a reactivation rate of 5.4E-4 per gen (½ main reactivation rate). (A) Solid lines represent the number of genomes in each compartment over time (active = black, latent = red). Dashed lines represent the mean number of lineages in each compartment over time (active = black, latent = red). Arrows represent alignment sampling events (active = purple shades earlier and yellow shades later, latent = red). Grey shading indicates therapy. (B) Genetic distances from HX-B2 (nucleotide substitutions) of the sampled full-length (9719 bases) genomes (active = purple shades earlier and yellow shades later, latent = red). Grey shading indicates therapy. Grey shading indicates therapy. (C) Distance from the root of the phylogeny to each sequence (in nucleotide substitutions per site) versus collection time. Reservoir sequences appear as red triangles and active sequences appear as circles coloured by collection year (with purple shades earlier and yellow shades later). Grey shading indicates a period of suppressive therapy. (D) Tip-to-tip distances (in nucleotide substitutions per site) between active sequences (WA), between latent sequences (WL) and from active sequences to latent sequences (Between). (E) Rooted maximum likelihood phylogeny inferred from *nef* sequences of simulated data. Reservoir sequences appear as red triangles and active sequences appear as circles coloured by collection year (with purple shades earlier and yellow shades later). (F) Density plot of the integration dates (Real) and density plots of the estimated integration dates using each method. (G) Density plots of the error of estimating the integration each reservoir genome. CS: Closest Sequence, CD: Clade, LR: Linear Regression, LS: Least Squares, ML: Maximum Likelihood.

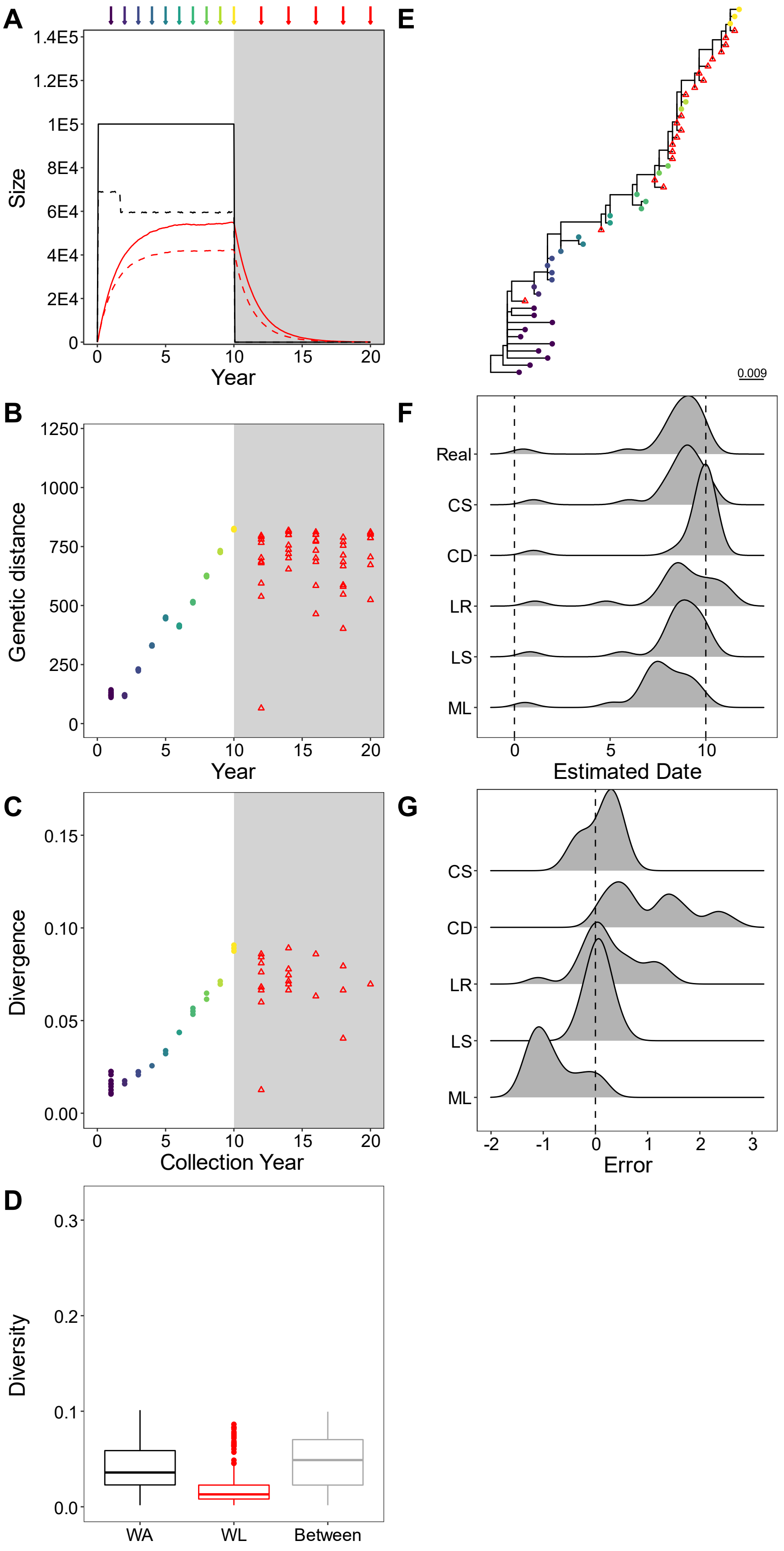

A
B
C
D
E
F
G
Size
Year
Genetic distance
Divergence
Collection Year
Diversity
WA
WL
Between
0.009
Real
CS
CD
LR
LS
ML
Estimated Date
Error

Supplementary Figure S11. Reactivation Rate (High). Simulated data set with a reactivation rate of 2.16E-3 per gen (2× main reactivation rate). (A) Solid lines represent the number of genomes in each compartment over time (active = black, latent = red). Dashed lines represent the mean number of lineages in each compartment over time (active = black, latent = red). Arrows represent alignment sampling events (active = purple shades earlier and yellow shades later, latent = red). Grey shading indicates therapy. (B) Genetic distances from HX-B2 (nucleotide substitutions) of the sampled full-length (9719 bases) genomes (active = purple shades earlier and yellow shades later, latent = red). Grey shading indicates therapy. Grey shading indicates therapy. (C) Distance from the root of the phylogeny to each sequence (in nucleotide substitutions per site) versus collection time. Reservoir sequences appear as red triangles and active sequences appear as circles coloured by collection year (with purple shades earlier and yellow shades later). Grey shading indicates a period of suppressive therapy. (D) Tip-to-tip distances (in nucleotide substitutions per site) between active sequences (WA), between latent sequences (WL) and from active sequences to latent sequences (Between). (E) Rooted maximum likelihood phylogeny inferred from *nef* sequences of simulated data. Reservoir sequences appear as red triangles and active sequences appear as circles coloured by collection year (with purple shades earlier and yellow shades later). (F) Density plot of the integration dates (Real) and density plots of the estimated integration dates using each method. (G) Density plots of the error of estimating the integration each reservoir genome. CS: Closest Sequence, CD: Clade, LR: Linear Regression, LS: Least Squares, ML: Maximum Likelihood.

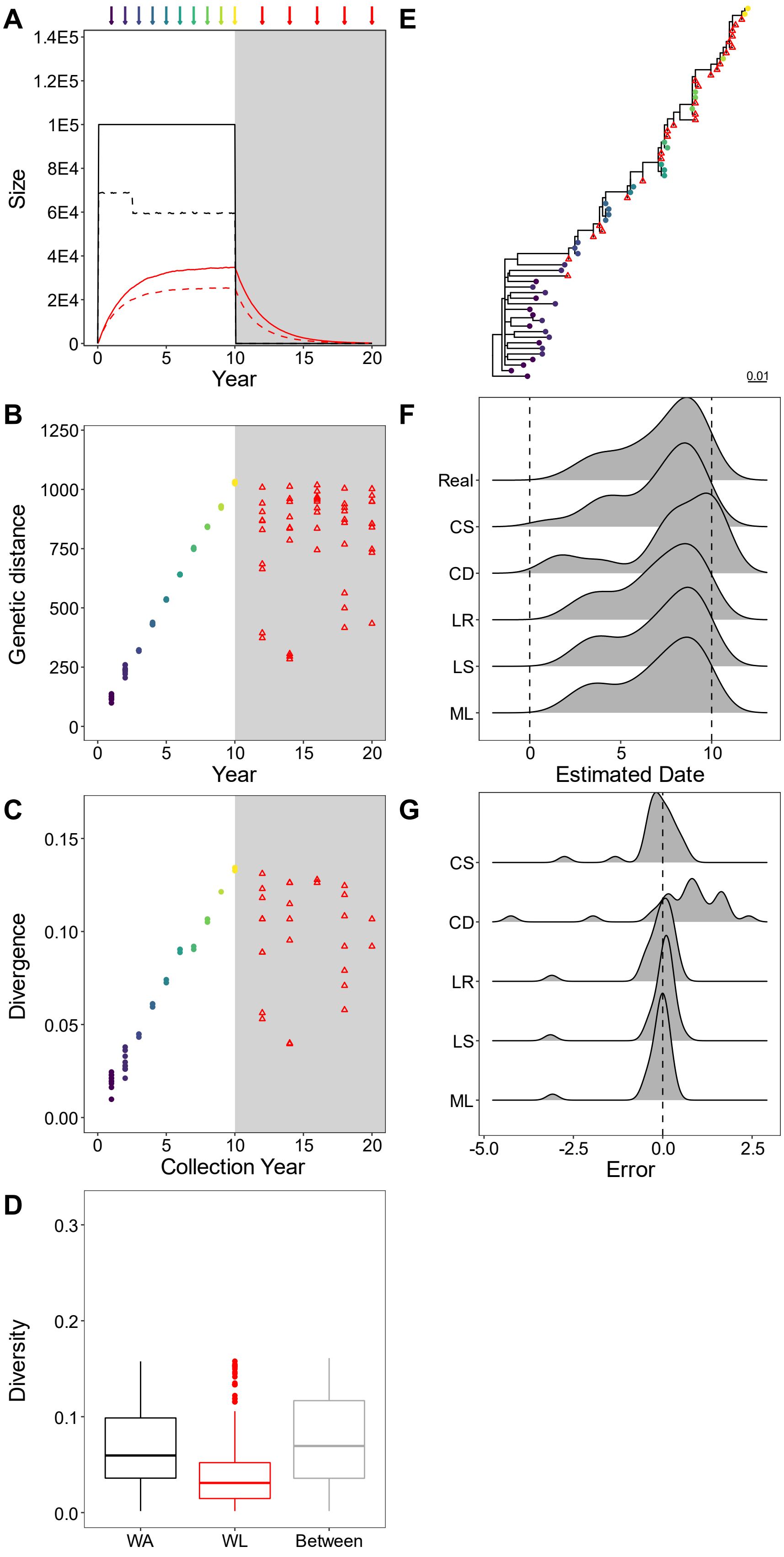

A
B
C
D
E
F
G
Size
Year
Genetic distance
Divergence
Collection Year
Diversity
WA
WL
Between
0.01
Real
CS
CD
LR
LS
ML
Estimated Date
Error

Supplementary Figure S12. Latency Rate (Low). Simulated data set with a latency rate of 1.3E-3 per gen (½ main latency rate). (A) Solid lines represent the number of genomes in each compartment over time (active = black, latent = red). Dashed lines represent the mean number of lineages in each compartment over time (active = black, latent = red). Arrows represent alignment sampling events (active = purple shades earlier and yellow shades later, latent = red). Grey shading indicates therapy. (B) Genetic distances from HX-B2 (nucleotide substitutions) of the sampled full-length (9719 bases) genomes (active = purple shades earlier and yellow shades later, latent = red). Grey shading indicates therapy. Grey shading indicates therapy. (C) Distance from the root of the phylogeny to each sequence (in nucleotide substitutions per site) versus collection time. Reservoir sequences appear as red triangles and active sequences appear as circles coloured by collection year (with purple shades earlier and yellow shades later). Grey shading indicates a period of suppressive therapy. (D) Tip-to-tip distances (in nucleotide substitutions per site) between active sequences (WA), between latent sequences (WL) and from active sequences to latent sequences (Between). (E) Rooted maximum likelihood phylogeny inferred from *nef* sequences of simulated data. Reservoir sequences appear as red triangles and active sequences appear as circles coloured by collection year (with purple shades earlier and yellow shades later). (F) Density plot of the integration dates (Real) and density plots of the estimated integration dates using each method. (G) Density plots of the error of estimating the integration each reservoir genome. CS: Closest Sequence, CD: Clade, LR: Linear Regression, LS: Least Squares, ML: Maximum Likelihood.

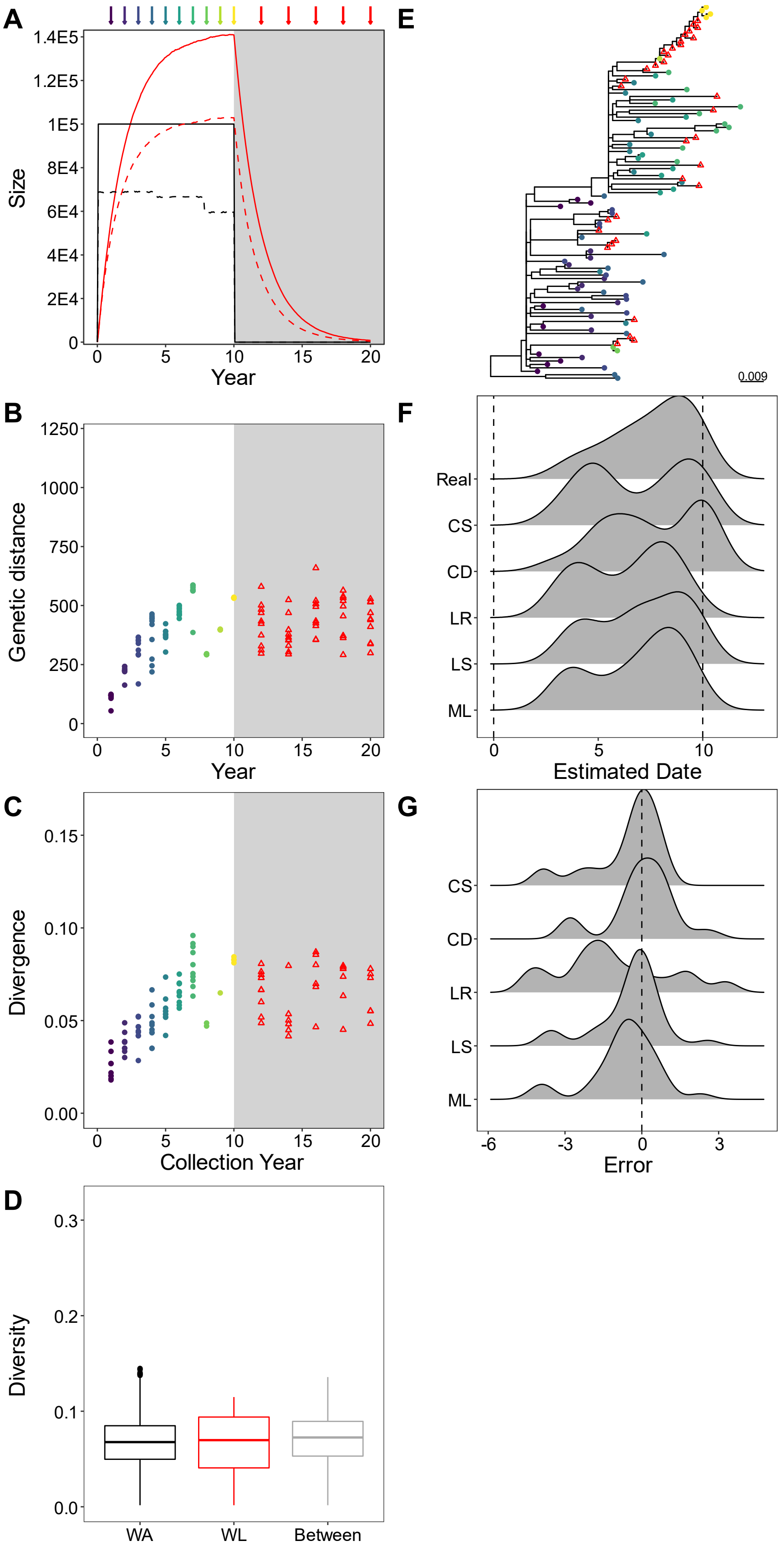

A
1.4E5
1.2E5
1E5
8E4
6E4
4E4
2E4
0
Size
0
5
10
15
20
Year
B
1250
1000
750
500
250
0
Genetic distance
Year
C
0.15
0.10
0.05
0.00
Divergence
Collection Year
D
0.3
0.2
0.1
0.0
Diversity
WA
WL
Between
E
0.009
F
Real
CS
CD
LR
LS
ML
Estimated Date
G
-6
-3
3
Error

Supplementary Figure S13. Latency Rate (High). Simulated data set with a latency rate of 5.2E-3 per gen (2× main latency rate). (A) Solid lines represent the number of genomes in each compartment over time (active = black, latent = red). Dashed lines represent the mean number of lineages in each compartment over time (active = black, latent = red). Arrows represent alignment sampling events (active = purple shades earlier and yellow shades later, latent = red). Grey shading indicates therapy. (B) Genetic distances from HX-B2 (nucleotide substitutions) of the sampled full-length (9719 bases) genomes (active = purple shades earlier and yellow shades later, latent = red). Grey shading indicates therapy. Grey shading indicates therapy. (C) Distance from the root of the phylogeny to each sequence (in nucleotide substitutions per site) versus collection time. Reservoir sequences appear as red triangles and active sequences appear as circles coloured by collection year (with purple shades earlier and yellow shades later). Grey shading indicates a period of suppressive therapy. (D) Tip-to-tip distances (in nucleotide substitutions per site) between active sequences (WA), between latent sequences (WL) and from active sequences to latent sequences (Between). (E) Rooted maximum likelihood phylogeny inferred from *nef* sequences of simulated data. Reservoir sequences appear as red triangles and active sequences appear as circles coloured by collection year (with purple shades earlier and yellow shades later). (F) Density plot of the integration dates (Real) and density plots of the estimated integration dates using each method. (G) Density plots of the error of estimating the integration each reservoir genome. CS: Closest Sequence, CD: Clade, LR: Linear Regression, LS: Least Squares, ML: Maximum Likelihood.

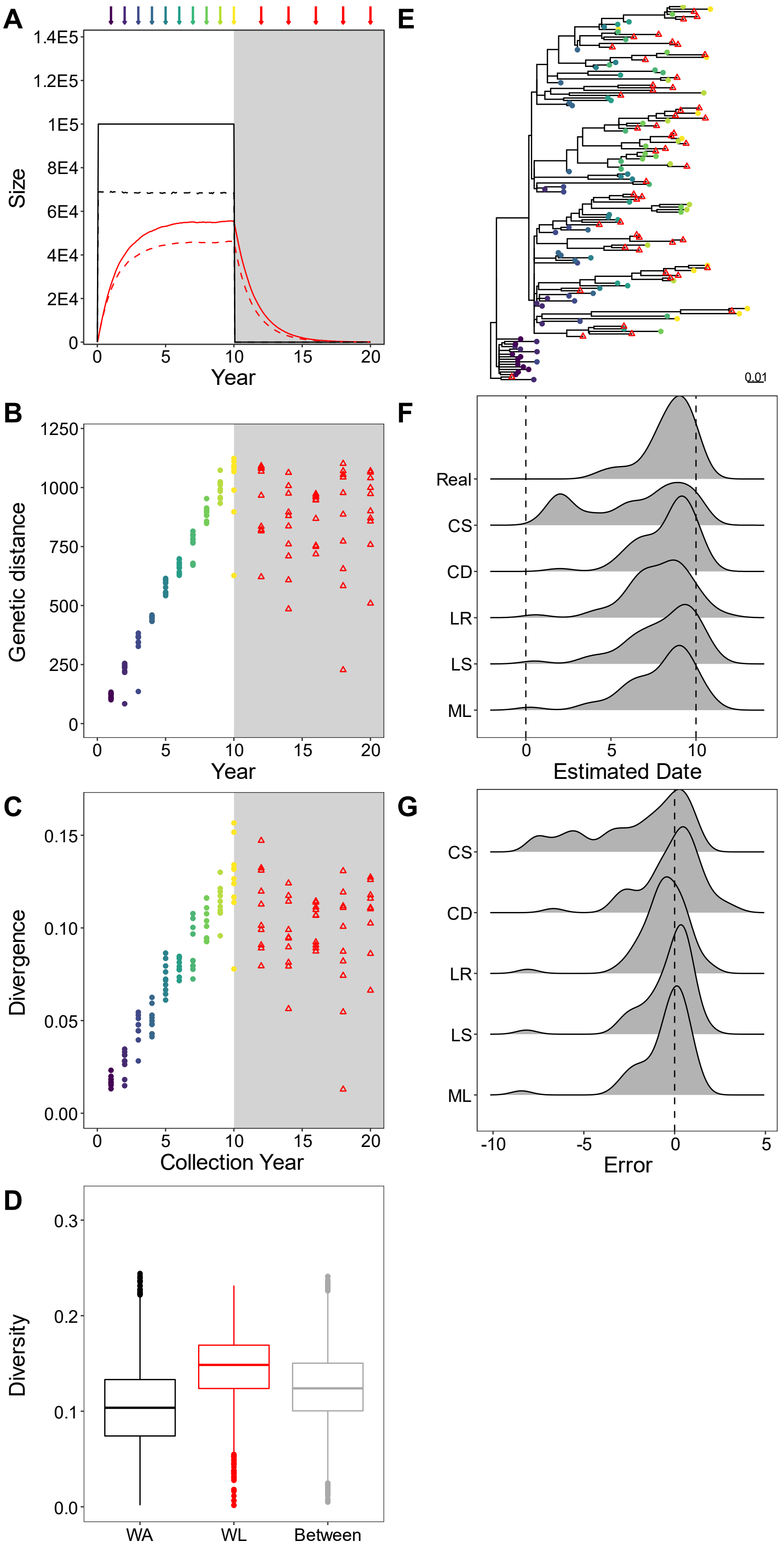

A
1.4E5
1.2E5
1E5
8E4
6E4
4E4
2E4
0
Size
0
5
10
15
20
Year
B
1250
1000
750
500
250
0
Genetic distance
Year
C
0.15
0.10
0.05
0.00
Divergence
Collection Year
D
0.3
0.2
0.1
0.0
Diversity
WA
WL
Between
E
0.01
F
Real
CS
CD
LR
LS
ML
Estimated Date
G
CS
CD
LR
LS
ML
-10
-5
0
5
Error

Supplementary Figure S14. Latent Growth Rate (Low). Simulated data set with a latent growth rate of 2E-3 per gen (⅔ main latent growth rate). (A) Solid lines represent the number of genomes in each compartment over time (active = black, latent = red). Dashed lines represent the mean number of lineages in each compartment over time (active = black, latent = red). Arrows represent alignment sampling events (active = purple shades earlier and yellow shades later, latent = red). Grey shading indicates therapy. (B) Genetic distances from HX-B2 (nucleotide substitutions) of the sampled full-length (9719 bases) genomes (active = purple shades earlier and yellow shades later, latent = red). Grey shading indicates therapy. Grey shading indicates therapy. (C) Distance from the root of the phylogeny to each sequence (in nucleotide substitutions per site) versus collection time. Reservoir sequences appear as red triangles and active sequences appear as circles coloured by collection year (with purple shades earlier and yellow shades later). Grey shading indicates a period of suppressive therapy. (D) Tip-to-tip distances (in nucleotide substitutions per site) between active sequences (WA), between latent sequences (WL) and from active sequences to latent sequences (Between). (E) Rooted maximum likelihood phylogeny inferred from *nef* sequences of simulated data. Reservoir sequences appear as red triangles and active sequences appear as circles coloured by collection year (with purple shades earlier and yellow shades later). (F) Density plot of the integration dates (Real) and density plots of the estimated integration dates using each method. (G) Density plots of the error of estimating the integration each reservoir genome. CS: Closest Sequence, CD: Clade, LR: Linear Regression, LS: Least Squares, ML: Maximum Likelihood.

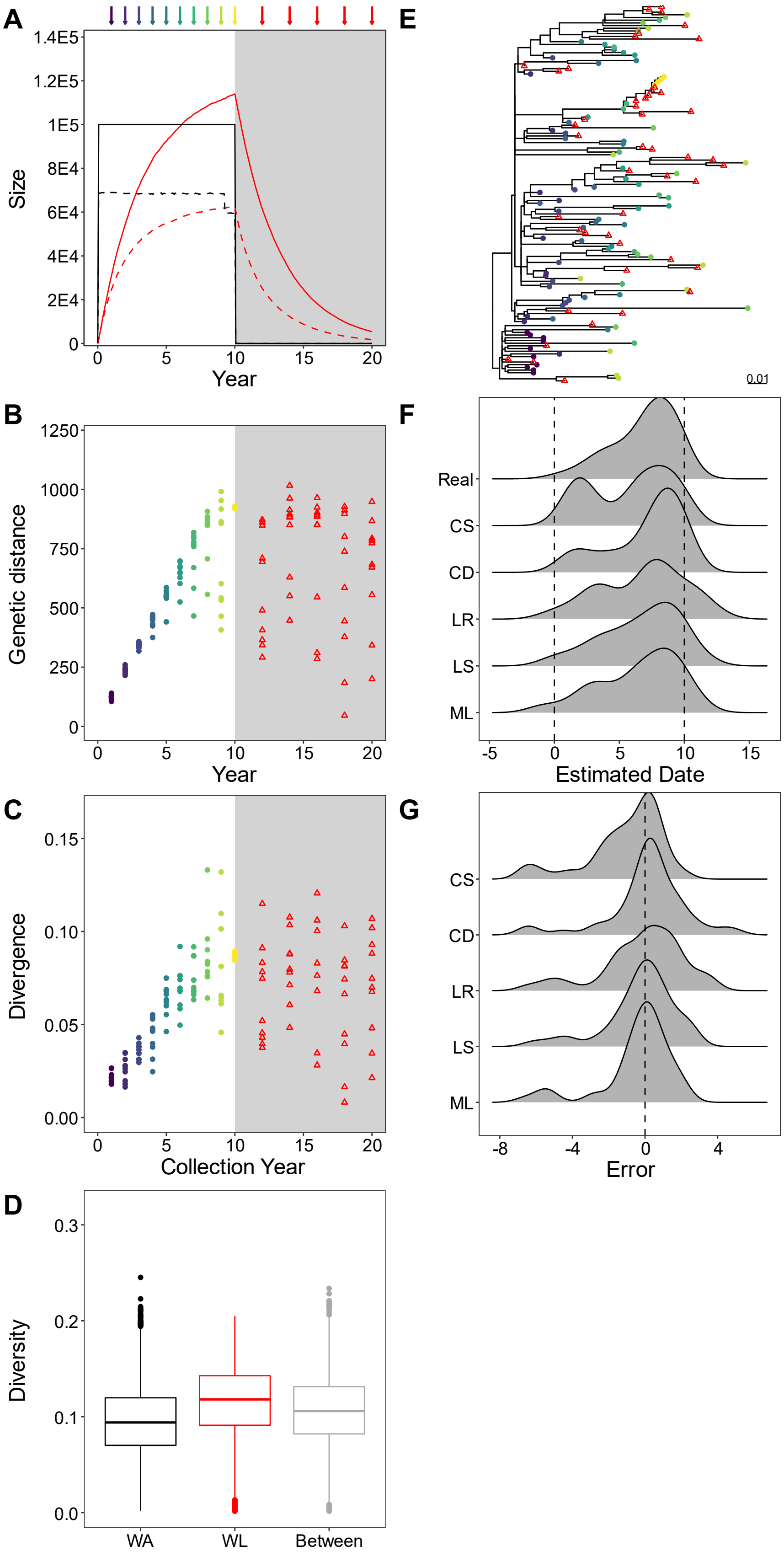
A
Size
1.4E5
1.2E5
1E5
8E4
6E4
4E4
2E4
0
Year
B
Genetic distance
1250
1000
750
500
250
C
Divergence
0.15
0.10
0.05
0.00
Collection Year
D
Diversity
0.3
0.2
0.1
0.0
WA
WL
Between
E
0.01
F
Real
CS
CD
LR
LS
ML
Estimated Date
G
Error

Supplementary Figure S15. Latent Growth Rate (High). Simulated data set with a latency growth rate of 4.5E-3 per gen (1.5× main latent growth rate). (A) Solid lines represent the number of genomes in each compartment over time (active = black, latent = red). Dashed lines represent the mean number of lineages in each compartment over time (active = black, latent = red). Arrows represent alignment sampling events (active = purple shades earlier and yellow shades later, latent = red). Grey shading indicates therapy. (B) Genetic distances from HX-B2 (nucleotide substitutions) of the sampled full-length (9719 bases) genomes (active = purple shades earlier and yellow shades later, latent = red). Grey shading indicates therapy. Grey shading indicates therapy. (C) Distance from the root of the phylogeny to each sequence (in nucleotide substitutions per site) versus collection time. Reservoir sequences appear as red triangles and active sequences appear as circles coloured by collection year (with purple shades earlier and yellow shades later). Grey shading indicates a period of suppressive therapy. (D) Tip-to-tip distances (in nucleotide substitutions per site) between active sequences (WA), between latent sequences (WL) and from active sequences to latent sequences (Between). (E) Rooted maximum likelihood phylogeny inferred from *nef* sequences of simulated data. Reservoir sequences appear as red triangles and active sequences appear as circles coloured by collection year (with purple shades earlier and yellow shades later). (F) Density plot of the integration dates (Real) and density plots of the estimated integration dates using each method. (G) Density plots of the error of estimating the integration each reservoir genome. CS: Closest Sequence, CD: Clade, LR: Linear Regression, LS: Least Squares, ML: Maximum Likelihood.

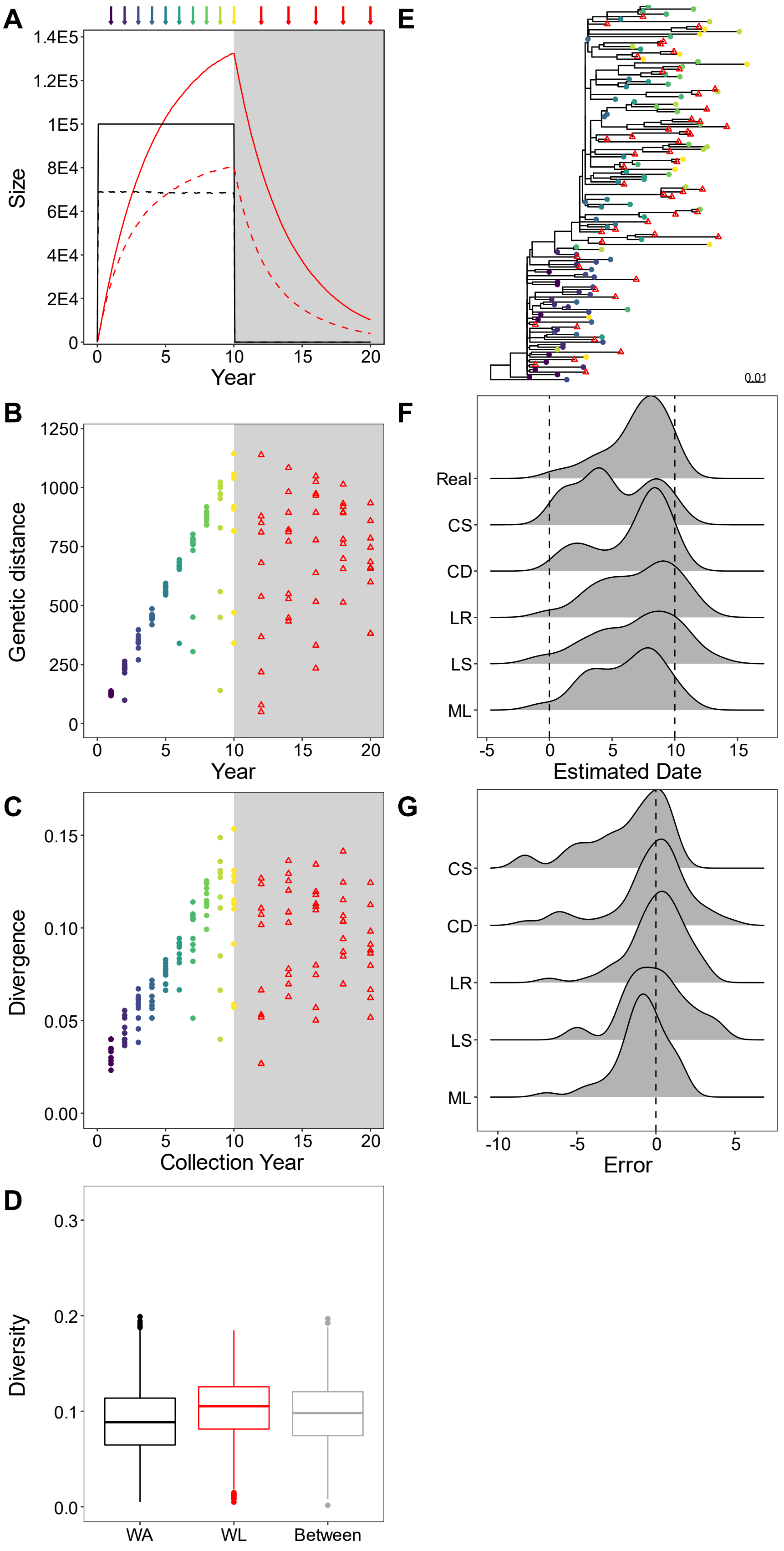

A
Size
Year
B
Genetic distance
Year
C
Divergence
Collection Year
D
Diversity
WA
WL
Between
E
0.01
F
Real
CS
CD
LR
LS
ML
Estimated Date
G
CS
CD
LR
LS
ML
Error

Supplementary Figure S16. Latent Death Rate (Low). Simulated data set with a latent death rate of 3.73E-3 per gen (⅔ main latent death rate). (A) Solid lines represent the number of genomes in each compartment over time (active = black, latent = red). Dashed lines represent the mean number of lineages in each compartment over time (active = black, latent = red). Arrows represent alignment sampling events (active = purple shades earlier and yellow shades later, latent = red). Grey shading indicates therapy. (B) Genetic distances from HX-B2 (nucleotide substitutions) of the sampled full-length (9719 bases) genomes (active = purple shades earlier and yellow shades later, latent = red). Grey shading indicates therapy. Grey shading indicates therapy. (C) Distance from the root of the phylogeny to each sequence (in nucleotide substitutions per site) versus collection time. Reservoir sequences appear as red triangles and active sequences appear as circles coloured by collection year (with purple shades earlier and yellow shades later). Grey shading indicates a period of suppressive therapy. (D) Tip-to-tip distances (in nucleotide substitutions per site) between active sequences (WA), between latent sequences (WL) and from active sequences to latent sequences (Between). (E) Rooted maximum likelihood phylogeny inferred from *nef* sequences of simulated data. Reservoir sequences appear as red triangles and active sequences appear as circles coloured by collection year (with purple shades earlier and yellow shades later). (F) Density plot of the integration dates (Real) and density plots of the estimated integration dates using each method. (G) Density plots of the error of estimating the integration each reservoir genome. CS: Closest Sequence, CD: Clade, LR: Linear Regression, LS: Least Squares, ML: Maximum Likelihood.

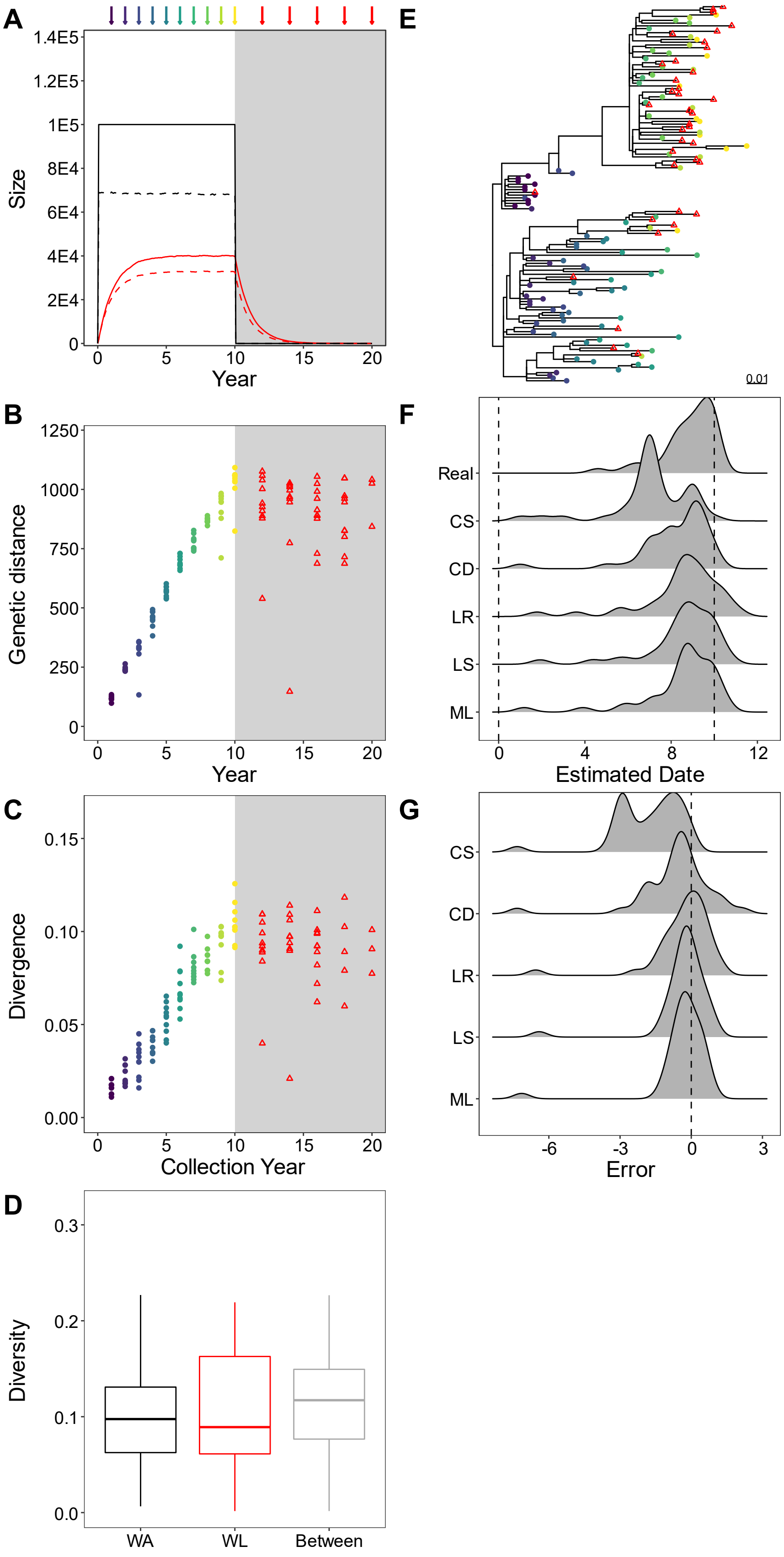
A
Size
Year
B
Genetic distance
Year
C
Divergence
Collection Year
D
Diversity
WA
WL
Between
E
0.01
F
Real
CS
CD
LR
LS
ML
Estimated Date
G
CS
CD
LR
LS
ML
Error

Supplementary Figure S17. Latent Death Rate (High). Simulated data set with a latent death rate of 8.4E-3 per gen (1.5× main latent death rate rate). (A) Solid lines represent the number of genomes in each compartment over time (active = black, latent = red). Dashed lines represent the mean number of lineages in each compartment over time (active = black, latent = red). Arrows represent alignment sampling events (active = purple shades earlier and yellow shades later, latent = red). Grey shading indicates therapy. (B) Genetic distances from HX-B2 (nucleotide substitutions) of the sampled full-length (9719 bases) genomes (active = purple shades earlier and yellow shades later, latent = red). Grey shading indicates therapy. Grey shading indicates therapy. (C) Distance from the root of the phylogeny to each sequence (in nucleotide substitutions per site) versus collection time. Reservoir sequences appear as red triangles and active sequences appear as circles coloured by collection year (with purple shades earlier and yellow shades later). Grey shading indicates a period of suppressive therapy. (D) Tip-to-tip distances (in nucleotide substitutions per site) between active sequences (WA), between latent sequences (WL) and from active sequences to latent sequences (Between). (E) Rooted maximum likelihood phylogeny inferred from *nef* sequences of simulated data. Reservoir sequences appear as red triangles and active sequences appear as circles coloured by collection year (with purple shades earlier and yellow shades later). (F) Density plot of the integration dates (Real) and density plots of the estimated integration dates using each method. (G) Density plots of the error of estimating the integration each reservoir genome. CS: Closest Sequence, CD: Clade, LR: Linear Regression, LS: Least Squares, ML: Maximum Likelihood.

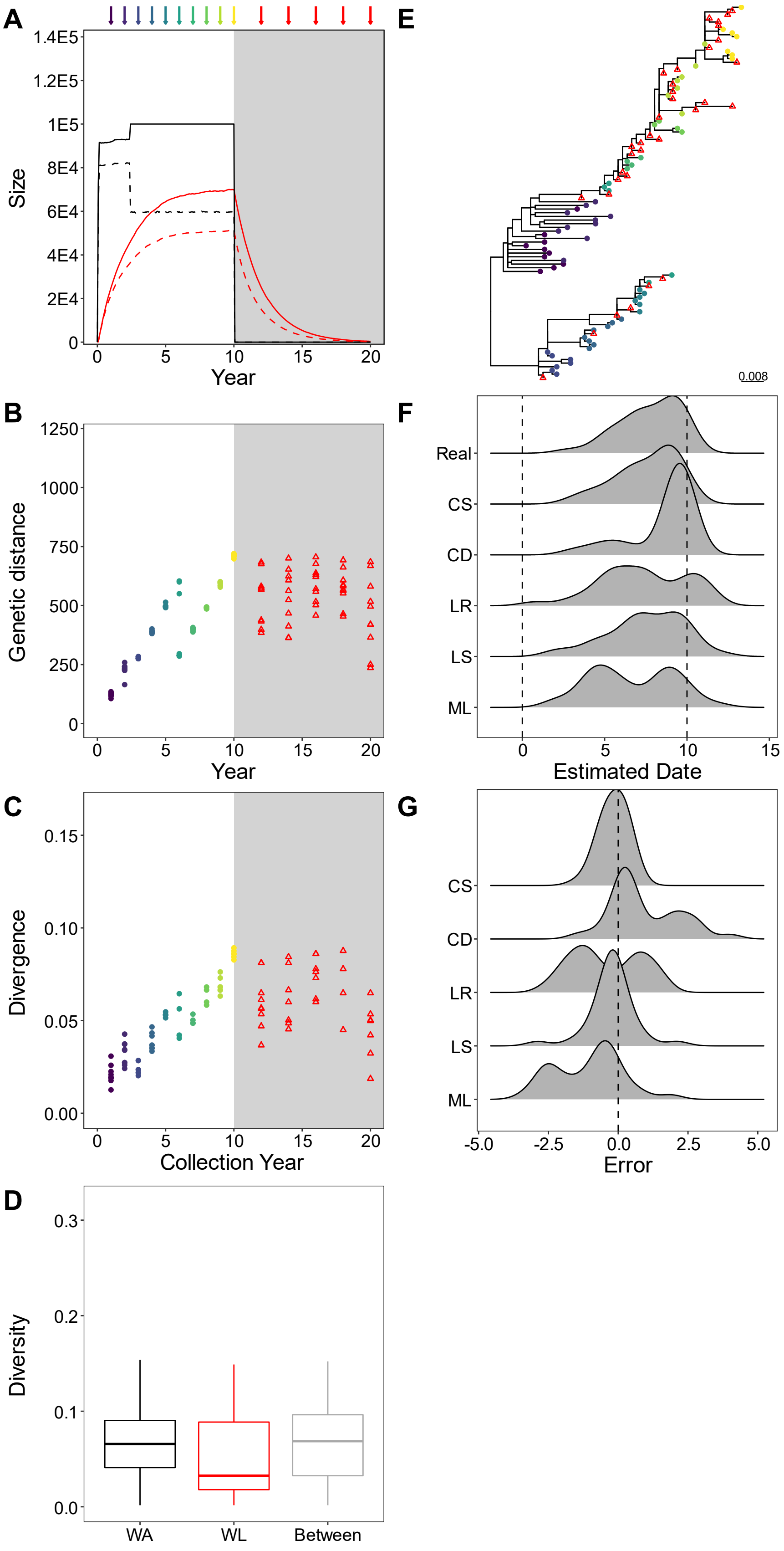

A
Size
Year
B
Genetic distance
Year
C
Divergence
Collection Year
D
Diversity
WA
WL
Between
E
0.008
F
Real
CS
CD
LR
LS
ML
Estimated Date
G
CS
CD
LR
LS
ML
Error

Supplementary Figure S18. Active Growth Rate (Low). Simulated data set with an active growth rate of 5 per gen (⅒ main active growth rate). (A) Solid lines represent the number of genomes in each compartment over time (active = black, latent = red). Dashed lines represent the mean number of lineages in each compartment over time (active = black, latent = red). Arrows represent alignment sampling events (active = purple shades earlier and yellow shades later, latent = red). Grey shading indicates therapy. (B) Genetic distances from HX-B2 (nucleotide substitutions) of the sampled full-length (9719 bases) genomes (active = purple shades earlier and yellow shades later, latent = red). Grey shading indicates therapy. Grey shading indicates therapy. (C) Distance from the root of the phylogeny to each sequence (in nucleotide substitutions per site) versus collection time. Reservoir sequences appear as red triangles and active sequences appear as circles coloured by collection year (with purple shades earlier and yellow shades later). Grey shading indicates a period of suppressive therapy. (D) Tip-to-tip distances (in nucleotide substitutions per site) between active sequences (WA), between latent sequences (WL) and from active sequences to latent sequences (Between). (E) Rooted maximum likelihood phylogeny inferred from *nef* sequences of simulated data. Reservoir sequences appear as red triangles and active sequences appear as circles coloured by collection year (with purple shades earlier and yellow shades later). (F) Density plot of the integration dates (Real) and density plots of the estimated integration dates using each method. (G) Density plots of the error of estimating the integration each reservoir genome. CS: Closest Sequence, CD: Clade, LR: Linear Regression, LS: Least Squares, ML: Maximum Likelihood.

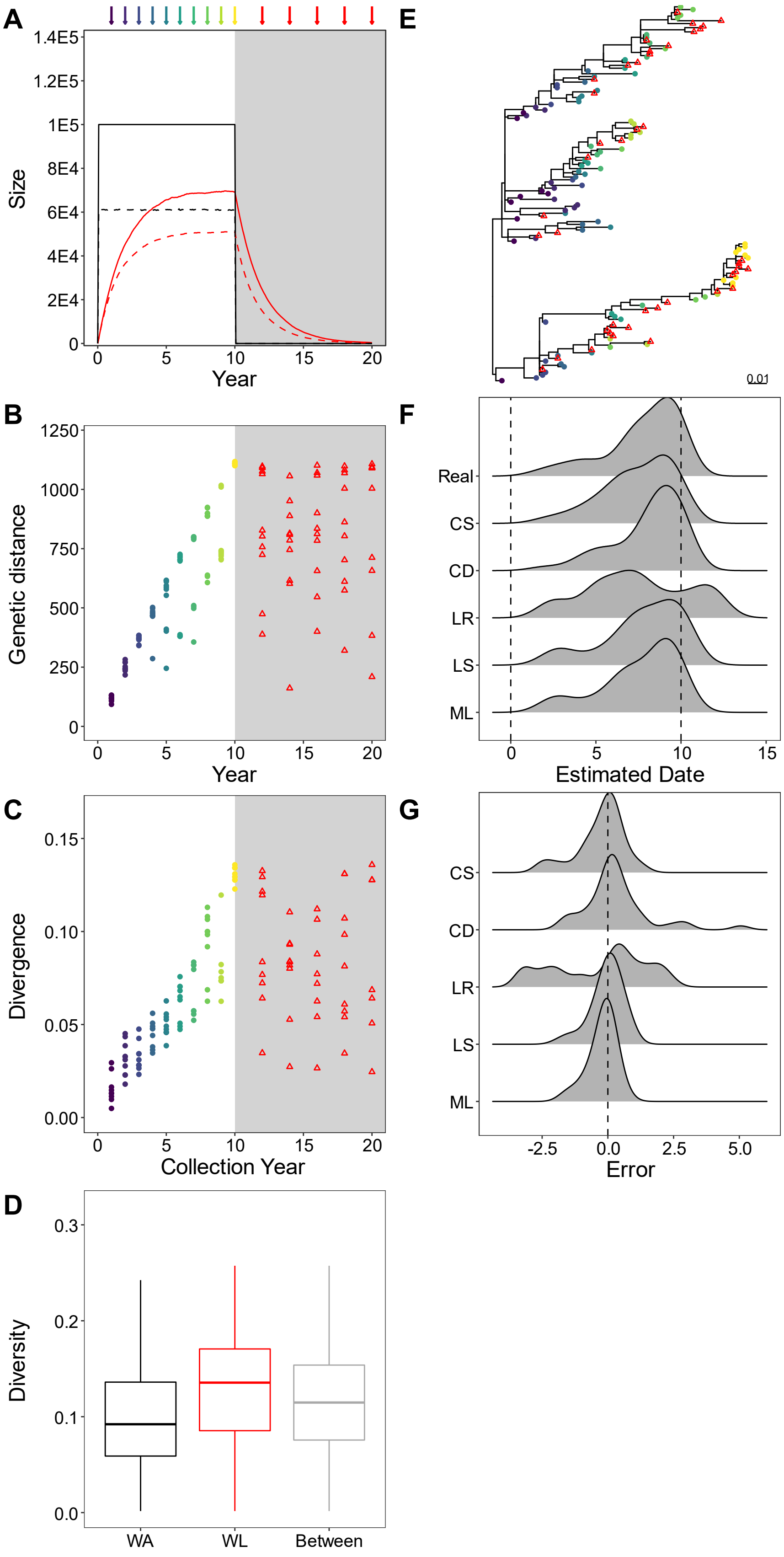
A
Size
Year
B
Genetic distance
Year
C
Divergence
Collection Year
D
Diversity
WA
WL
Between
E
0.01
F
Real
CS
CD
LR
LS
ML
Estimated Date
G
CS
CD
LR
LS
ML
Error

Supplementary Figure S19. Active Growth Rate (High). Simulated data set with an active growth rate of 500 per gen (10× main active growth rate). (A) Solid lines represent the number of genomes in each compartment over time (active = black, latent = red). Dashed lines represent the mean number of lineages in each compartment over time (active = black, latent = red). Arrows represent alignment sampling events (active = purple shades earlier and yellow shades later, latent = red). Grey shading indicates therapy. (B) Genetic distances from HX-B2 (nucleotide substitutions) of the sampled full-length (9719 bases) genomes (active = purple shades earlier and yellow shades later, latent = red). Grey shading indicates therapy. Grey shading indicates therapy. (C) Distance from the root of the phylogeny to each sequence (in nucleotide substitutions per site) versus collection time. Reservoir sequences appear as red triangles and active sequences appear as circles coloured by collection year (with purple shades earlier and yellow shades later). Grey shading indicates a period of suppressive therapy. (D) Tip-to-tip distances (in nucleotide substitutions per site) between active sequences (WA), between latent sequences (WL) and from active sequences to latent sequences (Between). (E) Rooted maximum likelihood phylogeny inferred from *nef* sequences of simulated data. Reservoir sequences appear as red triangles and active sequences appear as circles coloured by collection year (with purple shades earlier and yellow shades later). (F) Density plot of the integration dates (Real) and density plots of the estimated integration dates using each method. (G) Density plots of the error of estimating the integration each reservoir genome. CS: Closest Sequence, CD: Clade, LR: Linear Regression, LS: Least Squares, ML: Maximum Likelihood.

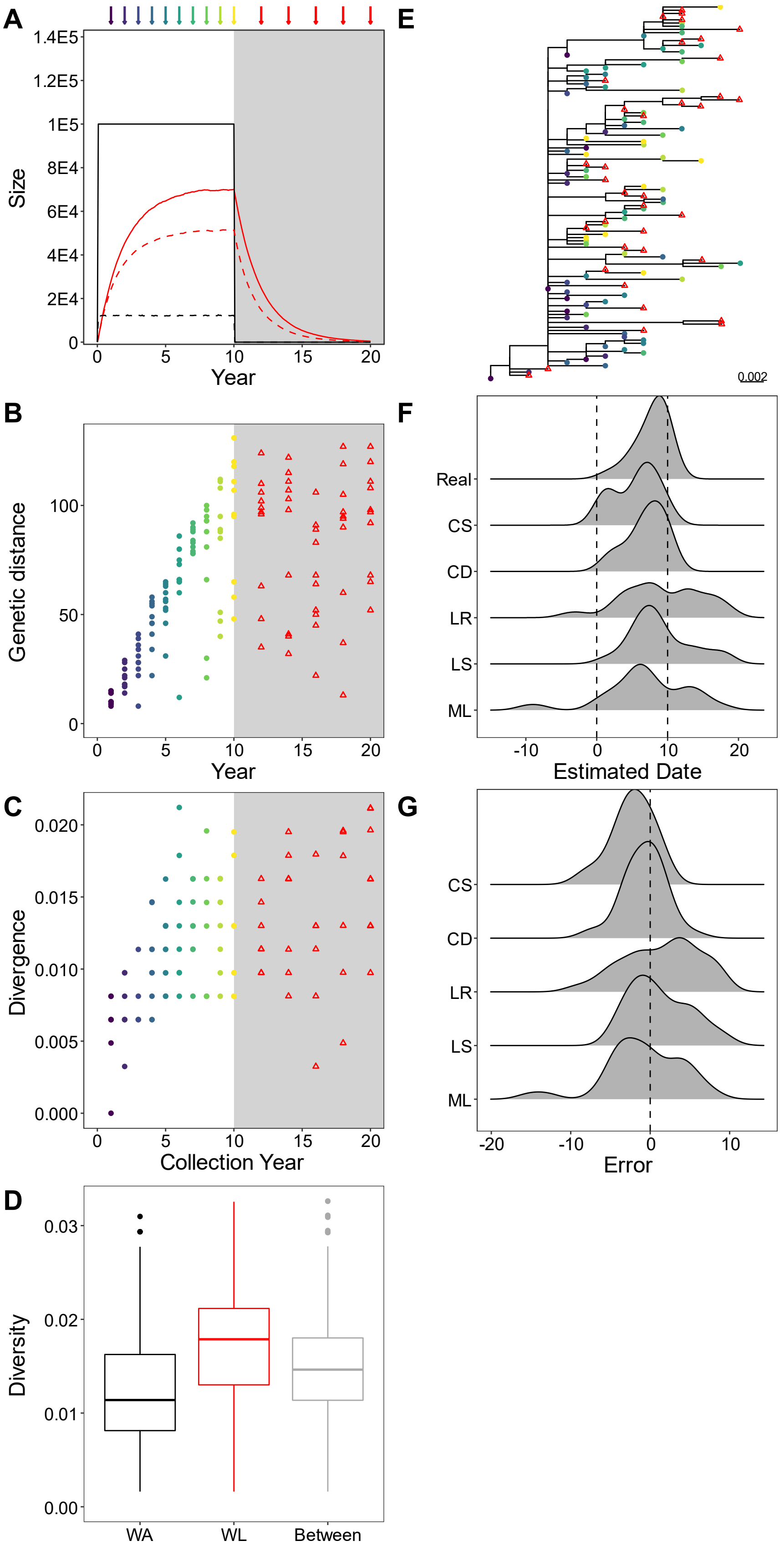

A
B
C
D
E
F
G
Size
Year
Genetic distance
Divergence
Collection Year
Diversity
WA
WL
Between
0.002
Real
CS
CD
LR
LS
ML
Estimated Date
Error

Supplementary Figure S20. Mutation Rate (Low). Simulated data set with a mutation rate of 9.3E-6 nucleotide substitutions per site per gen (⅒ main mutation rate). Note that the y axes have different scales than in other figures. (A) Solid lines represent the number of genomes in each compartment over time (active = black, latent = red). Dashed lines represent the mean number of lineages in each compartment over time (active = black, latent = red). Arrows represent alignment sampling events (active = purple shades earlier and yellow shades later, latent = red). Grey shading indicates therapy. (B) Genetic distances from HX-B2 (nucleotide substitutions) of the sampled full-length (9719 bases) genomes (active = purple shades earlier and yellow shades later, latent = red). Grey shading indicates therapy. Grey shading indicates therapy. (C) Distance from the root of the phylogeny to each sequence (in nucleotide substitutions per site) versus collection time. Reservoir sequences appear as red triangles and active sequences appear as circles coloured by collection year (with purple shades earlier and yellow shades later). Grey shading indicates a period of suppressive therapy. (D) Tip-to-tip distances (in nucleotide substitutions per site) between active sequences (WA), between latent sequences (WL) and from active sequences to latent sequences (Between). (E) Rooted maximum likelihood phylogeny inferred from *nef* sequences of simulated data. Reservoir sequences appear as red triangles and active sequences appear as circles coloured by collection year (with purple shades earlier and yellow shades later). (F) Density plot of the integration dates (Real) and density plots of the estimated integration dates using each method. (G) Density plots of the error of estimating the integration each reservoir genome. CS: Closest Sequence, CD: Clade, LR: Linear Regression, LS: Least Squares, ML: Maximum Likelihood.

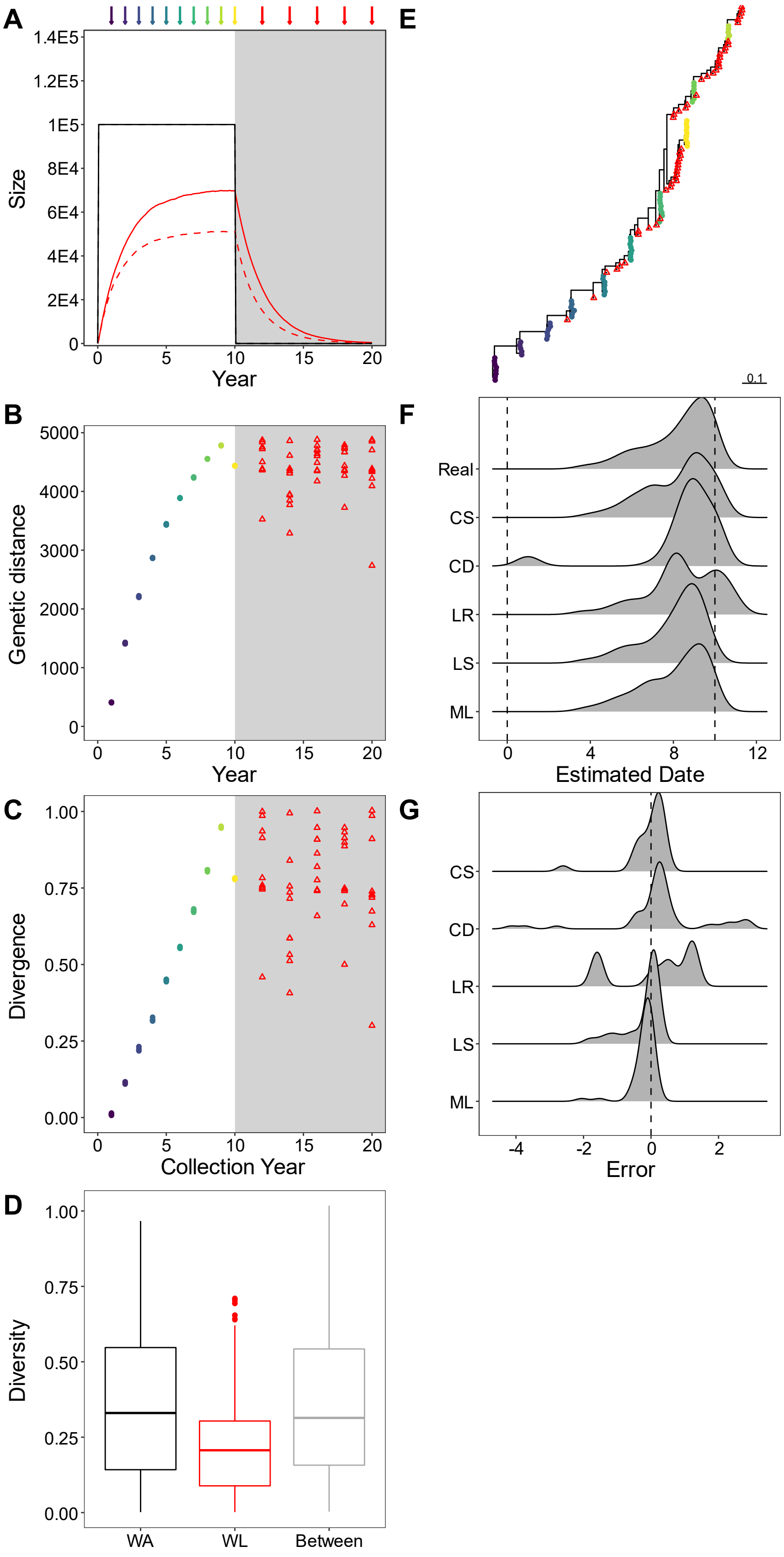

A
B
C
D
E
F
G
Size
Year
Genetic distance
Divergence
Collection Year
Diversity
WA
WL
Between
0.1
Real
CS
CD
LR
LS
ML
Estimated Date
Error

Supplementary Figure S21. Mutation Rate (High). Simulated data set with a latent death rate of 9.3E-4 nucleotide substitutions per site per gen (10× main reactivate rate). Note that the y axes have different scales than in other figures. (A) Solid lines represent the number of genomes in each compartment over time (active = black, latent = red). Dashed lines represent the mean number of lineages in each compartment over time (active = black, latent = red). Arrows represent alignment sampling events (active = purple shades earlier and yellow shades later, latent = red). Grey shading indicates therapy. (B) Genetic distances from HX-B2 (nucleotide substitutions) of the sampled full-length (9719 bases) genomes (active = purple shades earlier and yellow shades later, latent = red). Grey shading indicates therapy. Grey shading indicates therapy. (C) Distance from the root of the phylogeny to each sequence (in nucleotide substitutions per site) versus collection time. Reservoir sequences appear as red triangles and active sequences appear as circles coloured by collection year (with purple shades earlier and yellow shades later). Grey shading indicates a period of suppressive therapy. (D) Tip-to-tip distances (in nucleotide substitutions per site) between active sequences (WA), between latent sequences (WL) and from active sequences to latent sequences (Between). (E) Rooted maximum likelihood phylogeny inferred from *nef* sequences of simulated data. Reservoir sequences appear as red triangles and active sequences appear as circles coloured by collection year (with purple shades earlier and yellow shades later). (F) Density plot of the integration dates (Real) and density plots of the estimated integration dates using each method. (G) Density plots of the error of estimating the integration each reservoir genome. CS: Closest Sequence, CD: Clade, LR: Linear Regression, LS: Least Squares, ML: Maximum Likelihood.

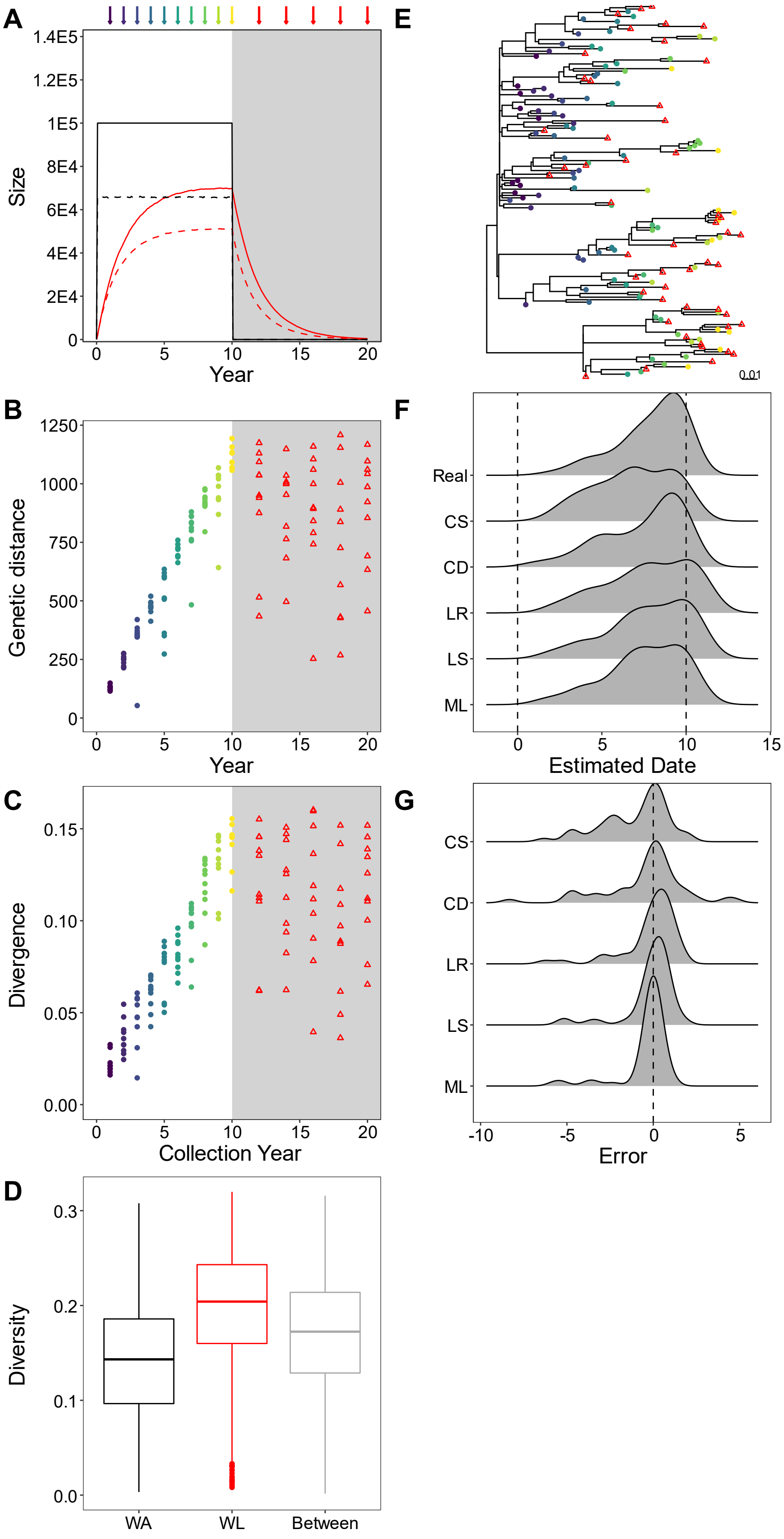
A
B
C
D
E
F
G
Size
Year
Genetic distance
Divergence
Collection Year
Diversity
WA
WL
Between
0.01
Real
CS
CD
LR
LS
ML
Estimated Date
Error

Supplementary Figure S22. Indel. Simulated data set with indels. The probability of a genome gaining an insertion or deletion was 6E-4 each and the size of the insertion or deletion was drawn from a negative binomial distribution with q=0.08 and r=1. These values were inferred from (Palmer and Y, 2019). Neutral fitness was imposed by removing all fitness specification including restrictions on coding regions (start and stop codons) because fitness interfered with indels in SANTA-SIM. The simulated genomes (and HXB2) were aligned using MAFFT v7.310 (Katoh and Standley, 2013) and clipped manually to the *nef* region in AliView v1.26 (Larsson, 2014). (A) Solid lines represent the number of genomes in each compartment over time (active = black, latent = red). Dashed lines represent the mean number of lineages in each compartment over time (active = black, latent = red). Arrows represent alignment sampling events (active = purple shades earlier and yellow shades later, latent = red). Grey shading indicates therapy. (B) Genetic distances from HX-B2 (nucleotide substitutions) of the sampled full-length (9719 bases) genomes (active = purple shades earlier and yellow shades later, latent = red). Grey shading indicates therapy. Grey shading indicates therapy. (C) Distance from the root of the phylogeny to each sequence (in nucleotide substitutions per site) versus collection time. Reservoir sequences appear as red triangles and active sequences appear as circles coloured by collection year (with purple shades earlier and yellow shades later). Grey shading indicates a period of suppressive therapy. (D) Tip-to-tip distances (in nucleotide substitutions per site) between active sequences (WA), between latent sequences (WL) and from active sequences to latent sequences (Between). (E) Rooted maximum likelihood phylogeny inferred from *nef* sequences of simulated data. Reservoir sequences appear as red triangles and active sequences appear as circles coloured by collection year (with purple shades earlier and yellow shades later). (F) Density plot of the integration dates (Real) and density plots of the estimated integration dates using each method. (G) Density plots of the error of estimating the integration each reservoir genome. CS: Closest Sequence, CD: Clade, LR: Linear Regression, LS: Least Squares, ML: Maximum Likelihood.

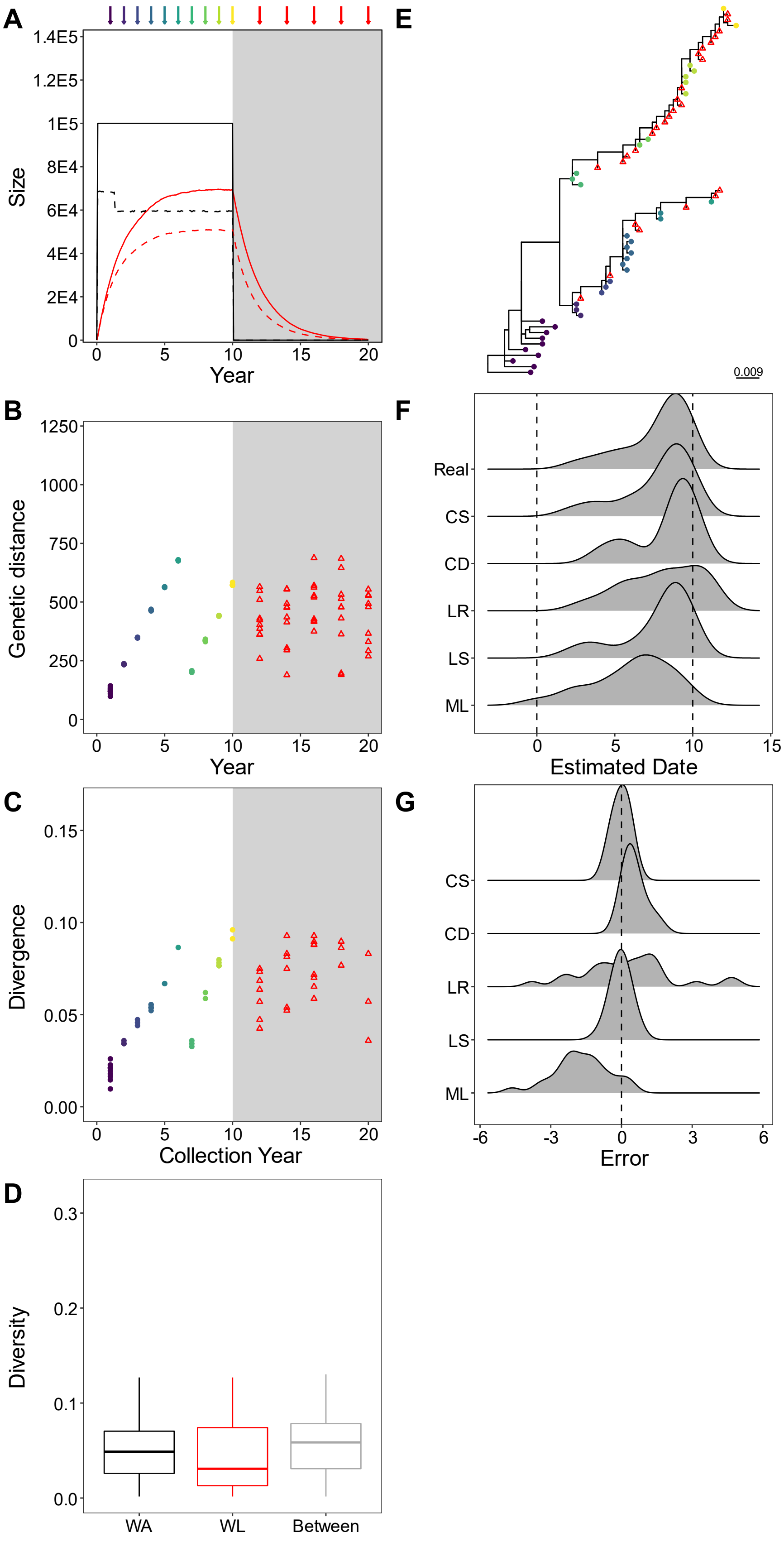

A
Size
1.4E5
1.2E5
1E5
8E4
6E4
4E4
2E4
0
Year
B
Genetic distance
1250
1000
750
500
250
Year
C
Divergence
0.15
0.10
0.05
0.00
Collection Year
D
Diversity
0.3
0.2
0.1
0.0
WA
WL
Between
E
0.009
F
Real
CS
CD
LR
LS
ML
Estimated Date
G
Error

Supplementary Figure S23. Recombination. Simulated data set with recombination. We chose a probability of 7% of any two genomes recombining and a probability of 1.4E-5 of recombination events happening at a particular site. These values were inferred from (Neher and Leitner, 2010). (A) Solid lines represent the number of genomes in each compartment over time (active = black, latent = red). Dashed lines represent the mean number of lineages in each compartment over time (active = black, latent = red). Arrows represent alignment sampling events (active = purple shades earlier and yellow shades later, latent = red). Grey shading indicates therapy. (B) Genetic distances from HX-B2 (nucleotide substitutions) of the sampled full-length (9719 bases) genomes (active = purple shades earlier and yellow shades later, latent = red). Grey shading indicates therapy. Grey shading indicates therapy. (C) Distance from the root of the phylogeny to each sequence (in nucleotide substitutions per site) versus collection time. Reservoir sequences appear as red triangles and active sequences appear as circles coloured by collection year (with purple shades earlier and yellow shades later). Grey shading indicates a period of suppressive therapy. (D) Tip-to-tip distances (in nucleotide substitutions per site) between active sequences (WA), between latent sequences (WL) and from active sequences to latent sequences (Between). (E) Rooted maximum likelihood phylogeny inferred from *nef* sequences of simulated data. Reservoir sequences appear as red triangles and active sequences appear as circles coloured by collection year (with purple shades earlier and yellow shades later). (F) Density plot of the integration dates (Real) and density plots of the estimated integration dates using each method. (G) Density plots of the error of estimating the integration each reservoir genome. CS: Closest Sequence, CD: Clade, LR: Linear Regression, LS: Least Squares, ML: Maximum Likelihood.

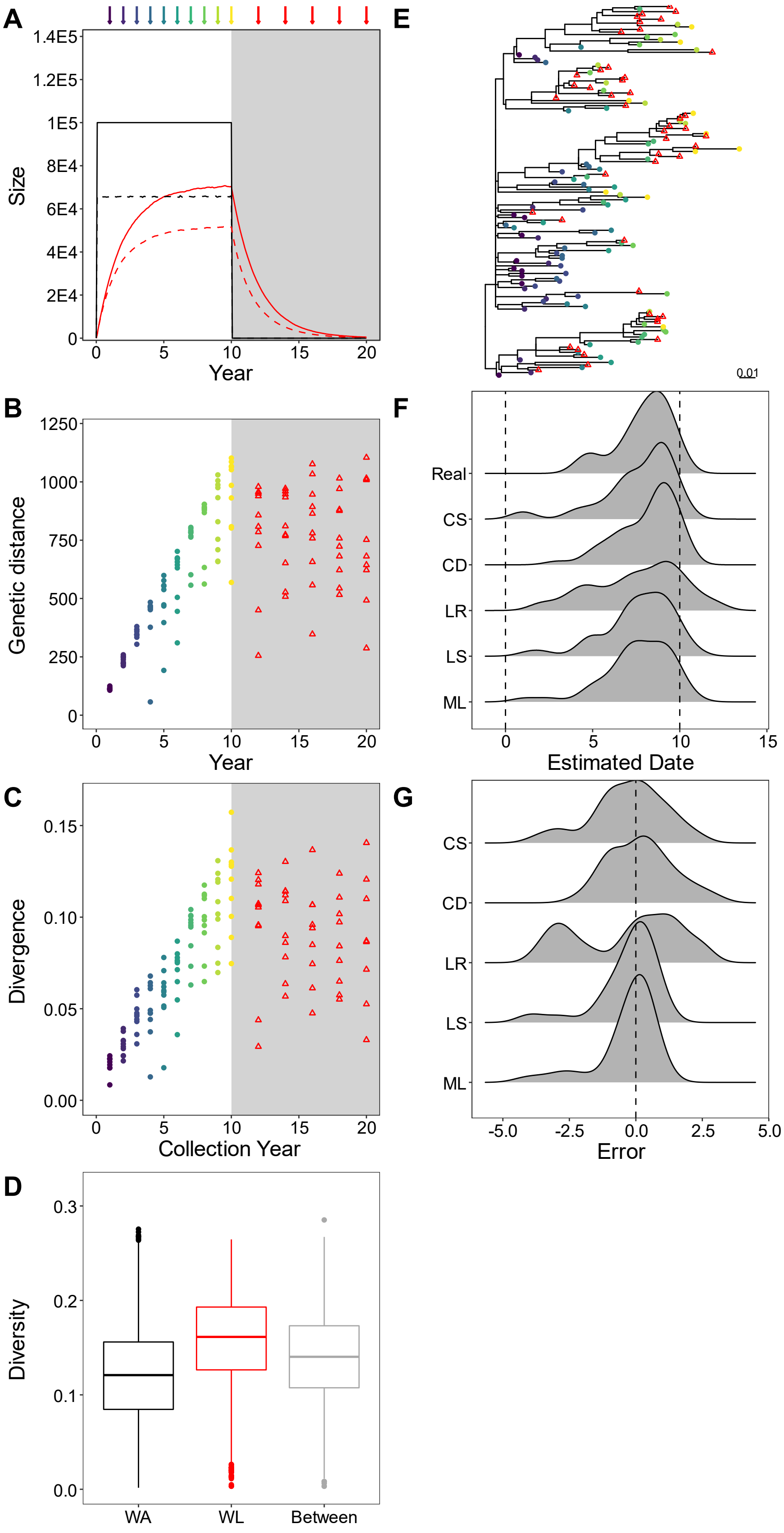
A
Size
Year
B
Genetic distance
Year
C
Divergence
Collection Year
D
Diversity
WA
WL
Between
E
0.01
F
Real
CS
CD
LR
LS
ML
Estimated Date
G
CS
CD
LR
LS
ML
Error

Supplementary Figure S24. Subtype C. Simulated data set with a HIV-1 subtype C starting virus. We used a full length length HIV-1 subtype C genome from Malawi (GenBank Accession KC156214) (Parrish et al., 2013) as starting genome in place of HXB2. We imposed neutral fitness, but with restrictions on coding regions. We did not allow stop codons in *gag*, *pol*, *vif*, *vpu*, *env*, and *nef*. Nonstop codons in *gag*, *vif*, *vpu*, *env*, and *nef* were given a fitness value of 0.001. (A) Solid lines represent the number of genomes in each compartment over time (active = black, latent = red). Dashed lines represent the mean number of lineages in each compartment over time (active = black, latent = red). Arrows represent alignment sampling events (active = purple shades earlier and yellow shades later, latent = red). Grey shading indicates therapy. (B) Genetic distances from HX-B2 (nucleotide substitutions) of the sampled full-length (9719 bases) genomes (active = purple shades earlier and yellow shades later, latent = red). Grey shading indicates therapy. Grey shading indicates therapy. (C) Distance from the root of the phylogeny to each sequence (in nucleotide substitutions per site) versus collection time. Reservoir sequences appear as red triangles and active sequences appear as circles coloured by collection year (with purple shades earlier and yellow shades later). Grey shading indicates a period of suppressive therapy. (D) Tip-to-tip distances (in nucleotide substitutions per site) between active sequences (WA), between latent sequences (WL) and from active sequences to latent sequences (Between). (E) Rooted maximum likelihood phylogeny inferred from *nef* sequences of simulated data. Reservoir sequences appear as red triangles and active sequences appear as circles coloured by collection year (with purple shades earlier and yellow shades later). (F) Density plot of the integration dates (Real) and density plots of the estimated integration dates using each method. (G) Density plots of the error of estimating the integration each reservoir genome. CS: Closest Sequence, CD: Clade, LR: Linear Regression, LS: Least Squares, ML: Maximum Likelihood.

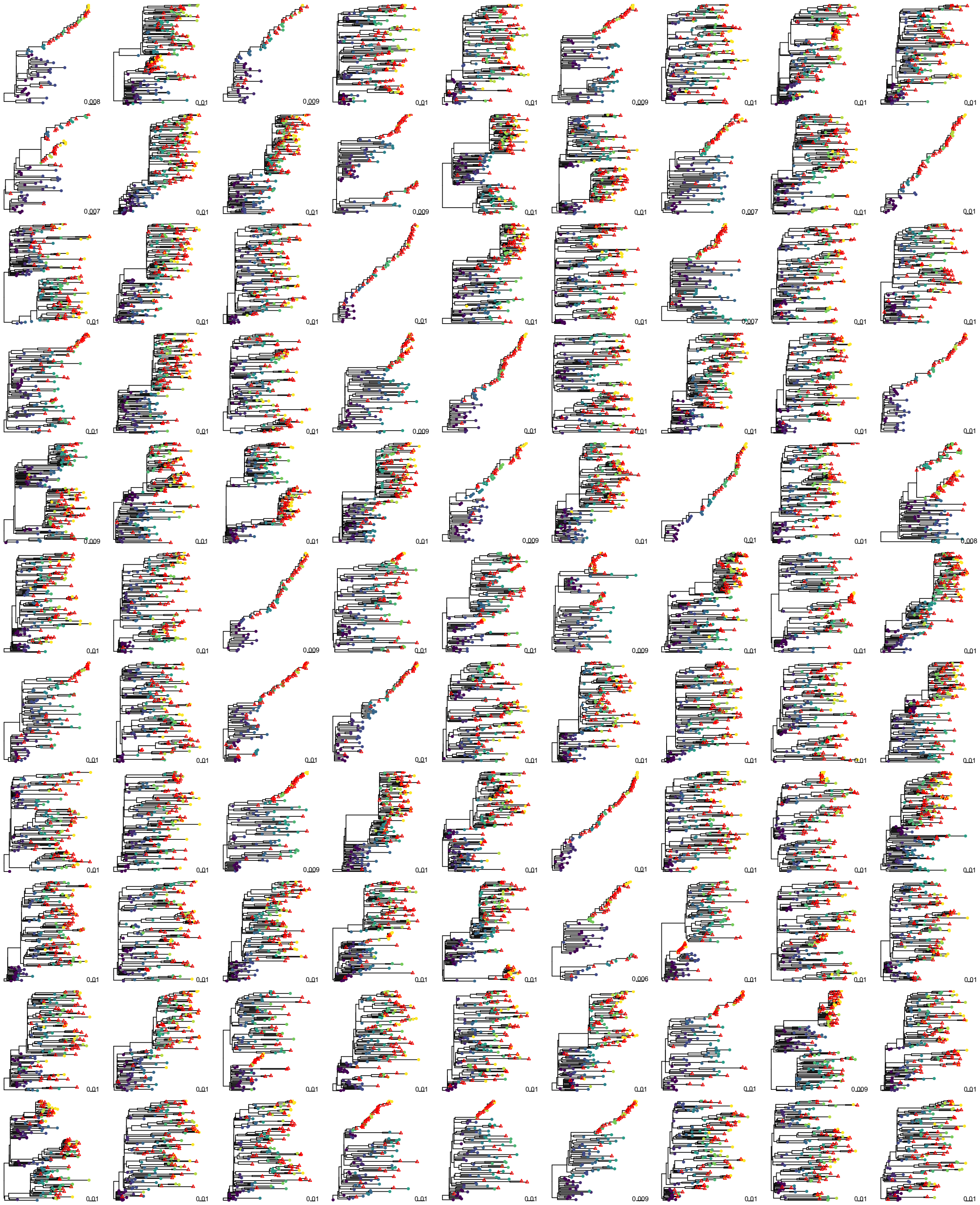

Supplementary Figure S25. Phylogenies of simulated data. Maximum likelihood phylogenies generated from the other 99 simulated data sets generated.

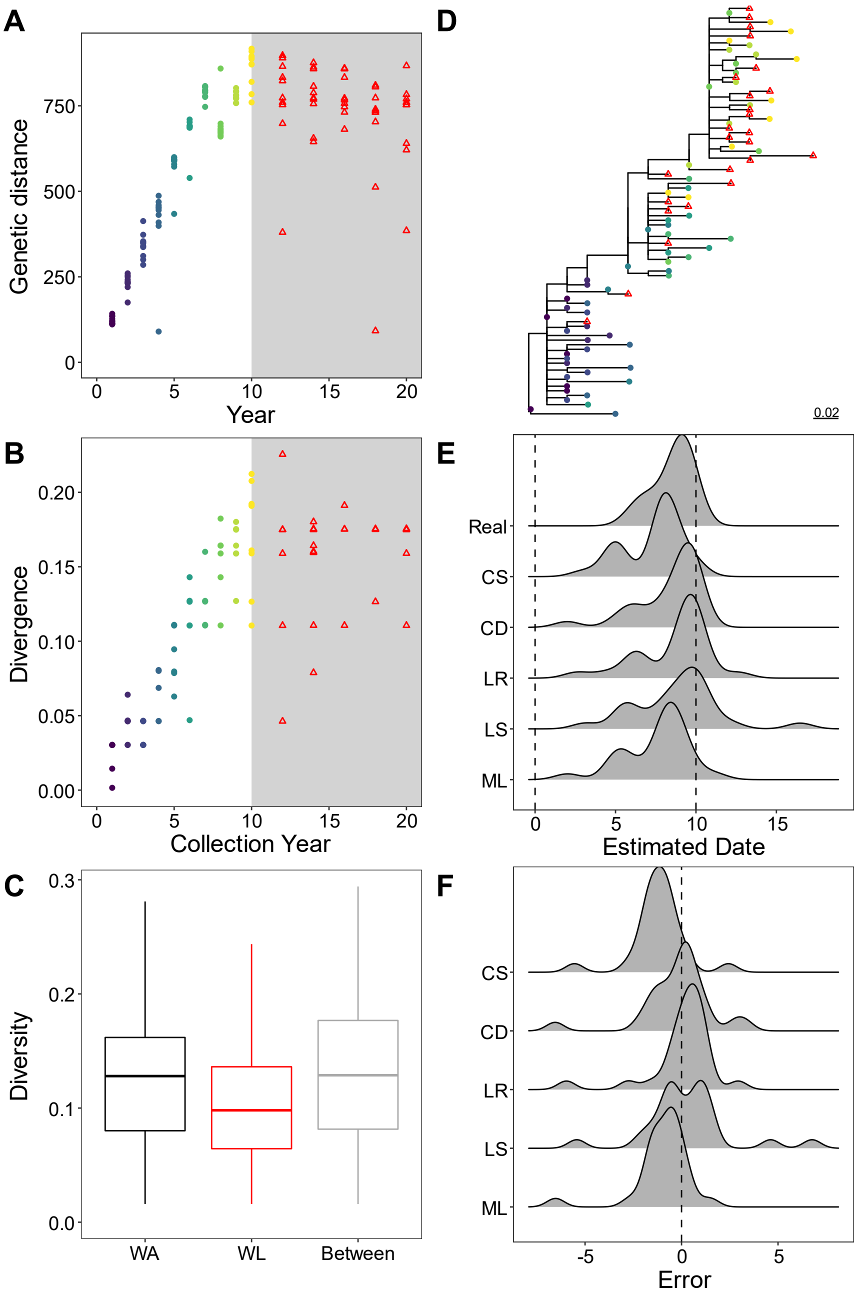

Supplementary Figure S26. Simulated data with the region of the genome from nucleotide positions 9,163 to 9,225 (*nef* codons: 123-143) used for phylogenetic inference and date estimation instead of full *nef*. The alignments were clipped manually in AliView v1.26 (Larsson, 2014). This is the same data set as in Supplementary Figure S2. Note that the y axes have different scales than in other figures. (A) Genetic distances from HX-B2 (nucleotide substitutions) of the sampled full-length (9719 bases) genomes (active = purple shades earlier and

yellow shades later, latent = red). Grey shading indicates therapy. Grey shading indicates therapy. (B) Distance from the root of the phylogeny to each sequence (in nucleotide substitutions per site) versus collection time. Reservoir sequences appear as red triangles and active sequences appear as circles coloured by collection year (with purple shades earlier and yellow shades later). Grey shading indicates a period of suppressive therapy. (C) Tip-to-tip distances (in nucleotide substitutions per site) between active sequences (WA), between latent sequences (WL) and from active sequences to latent sequences (Between). (D) Rooted maximum likelihood phylogeny inferred from *nef* sequences of simulated data. Reservoir sequences appear as red triangles and active sequences appear as circles coloured by collection year (with purple shades earlier and yellow shades later). (E) Density plot of the integration dates (Real) and density plots of the estimated integration dates using each method. (F) Density plots of the error of estimating the integration each reservoir genome. CS: Closest Sequence, CD: Clade, LR: Linear Regression, LS: Least Squares, ML: Maximum Likelihood.

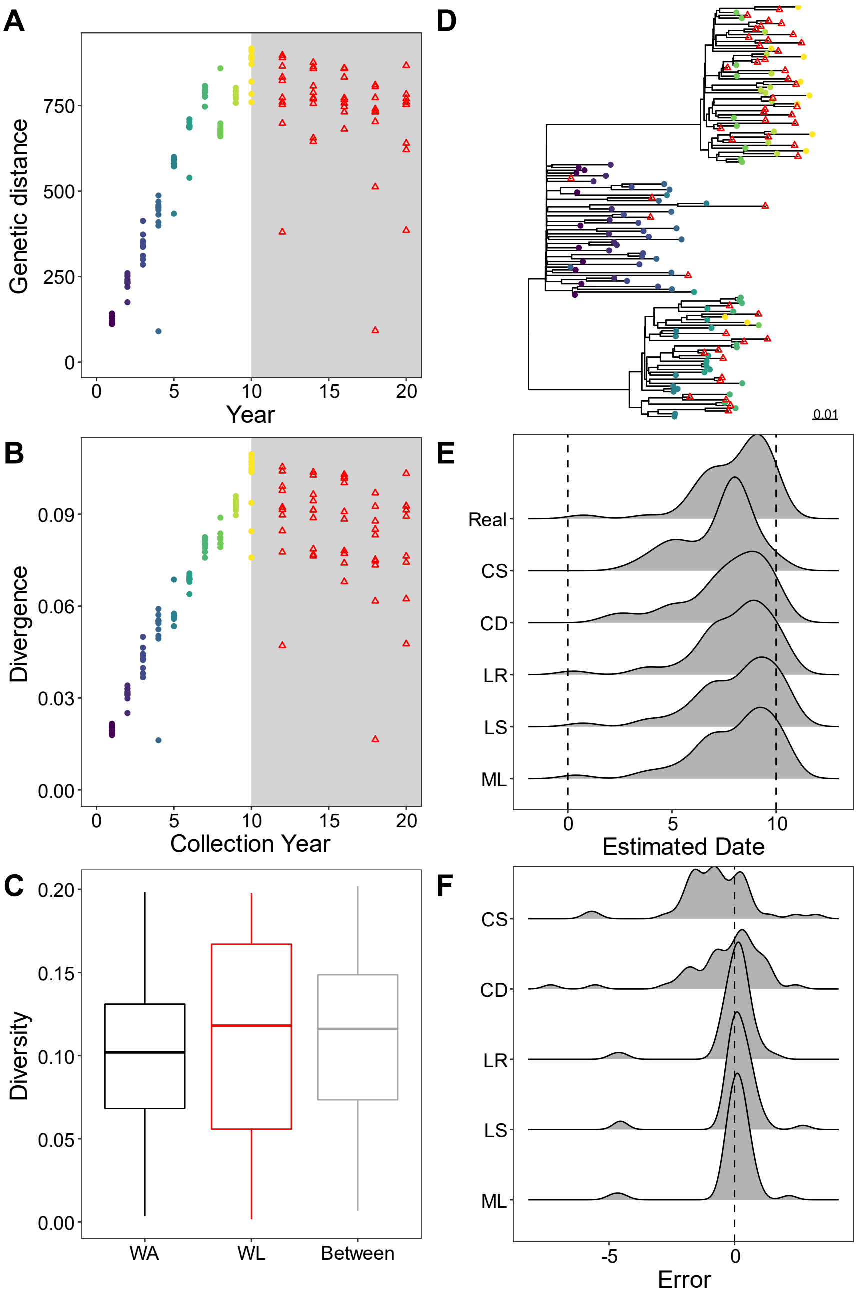

Supplementary Figure S27. Simulated data using the full genome for phylogenetic inference and date estimation instead of just *nef*. This is the same data set as in Supplementary Figure S2. Note that the y axes have different scales than in other figures. (A) Genetic distances from HX-B2 (nucleotide substitutions) of the sampled full-length (9719 bases) genomes (active = purple shades earlier and yellow shades later, latent = red). Grey shading indicates therapy. Grey shading indicates therapy. (B) Distance from the root of the phylogeny to each sequence (in nucleotide

substitutions per site) versus collection time. Reservoir sequences appear as red triangles and active sequences appear as circles coloured by collection year (with purple shades earlier and yellow shades later). Grey shading indicates a period of suppressive therapy. (C) Tip-to-tip distances (in nucleotide substitutions per site) between active sequences (WA), between latent sequences (WL) and from active sequences to latent sequences (Between). (D) Rooted maximum likelihood phylogeny inferred from *nef* sequences of simulated data. Reservoir sequences appear as red triangles and active sequences appear as circles coloured by collection year (with purple shades earlier and yellow shades later). (E) Density plot of the integration dates (Real) and density plots of the estimated integration dates using each method. (F) Density plots of the error of estimating the integration each reservoir genome. CS: Closest Sequence, CD: Clade, LR: Linear Regression, LS: Least Squares, ML: Maximum Likelihood.

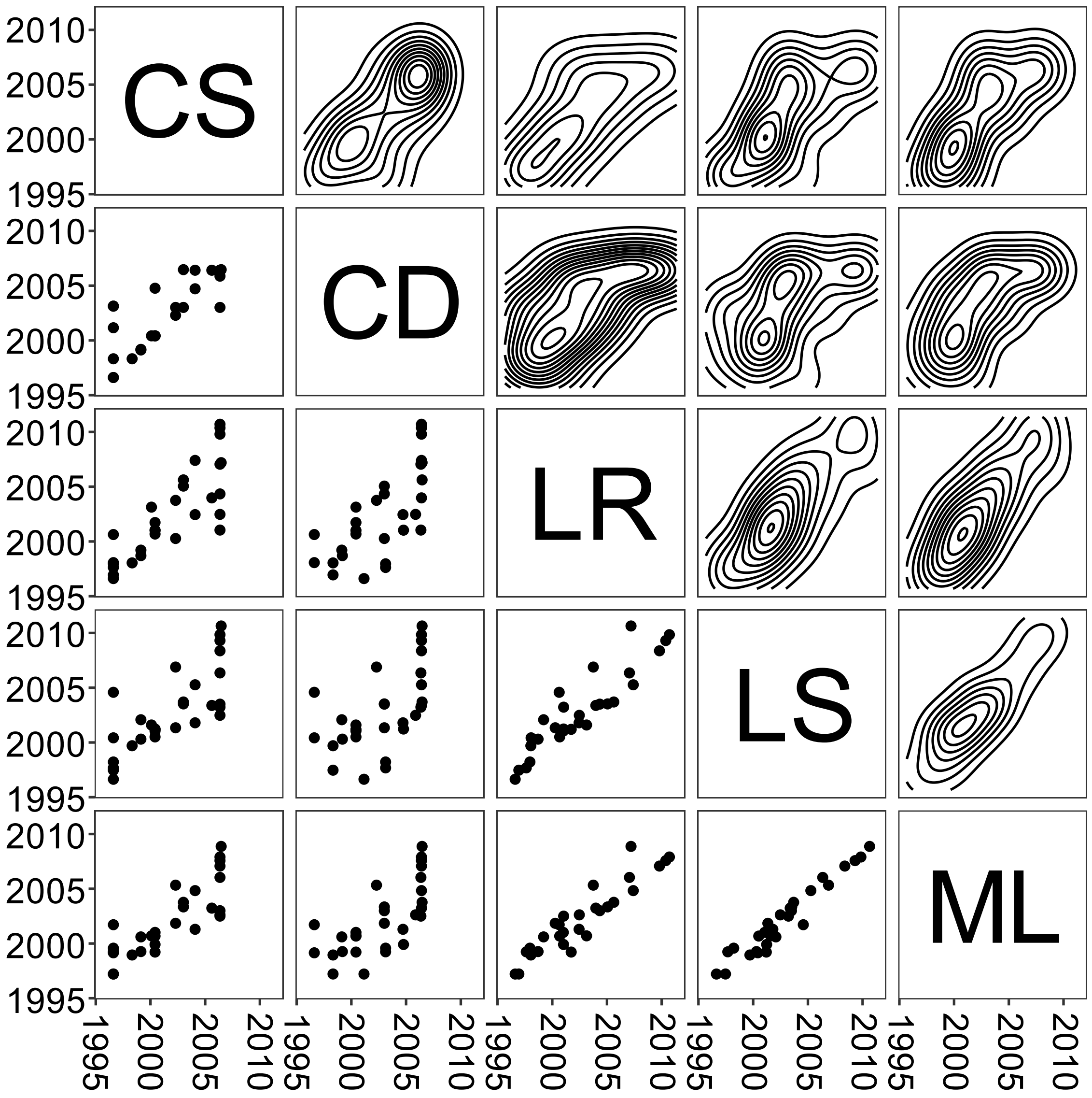

Supplementary Figure S28. Comparison of date estimation methods using empirical dating. In the bottom left pointwise estimates of sequences from empirical proviral DNA are compared between methods. In the top right 2d density plots of the estimate of sequences from empirical proviral DNA are shown comparing the methods. CS: Closest Sequence, CD: Clade, LR: Linear Regression, LS: Least Squares, ML: Maximum Likelihood

References

Barton, J.P. et al. (2019) ‘Modelling and in vitro testing of the HIV-1 Nef fitness landscape’, *Virus Evolution*, 5: a2vez029.

Brumme, Z.L. et al. (2009) ‘HLA-Associated Immune Escape Pathways in HIV-1 Subtype B Gag, Pol and Nef Proteins’, *Plos One*, 4: a2e6687.

Katoh, K., Standley, D.M. (2013) ‘MAFFT multiple sequence alignment software version 7: improvements in performance and usability’, *Mol Biol Evol*, 30: 772-780.

Larsson, A. (2014) ‘ AliView: a fast and lightweight alignment viewer and editor for large datasets’, *Bioinformatics*, 30: 3276-3278.

Neher, R.A., Leitner, T. (2010) ‘Recombination Rate and Selection Strength in HIV Intra-patient Evolution’, *Plos Comp Biol*, 6: a2e1000660.

Palmer, J., Y, P.A.F. (2019) ‘Phylogenetic measures of indel rate variation among the HIV-1 group M subtypes’, *Virus Evol*, 5: a2vez022.

Parrish, N.F. et al. (2013) ‘Phenotypic properties of transmitted founder HIV-1’, *Proc Natl Acad Sci U S A*, 110: 6626-6633.
